# Synthesis and Photocatalytic Performance of a Ferrite-Based Tungstate Nanocomposite for Imidacloprid Removal

**DOI:** 10.3390/nano16120721

**Published:** 2026-06-11

**Authors:** Irum Jamil, Abdulaziz Alasiri, Faisal Nawaz, Muqdssa Rashid, Abdullah A. Elfar, Md Enamul Hoque

**Affiliations:** 1 Department of Chemistry, University of Wah, Quaid Avenue, Rawalpindi 47040, Punjab, Pakistan; uw-22-chm-phd-003@student.uow.edu.pk (I.J.); uw-23-ochm-ms-004@student.uow.edu.pk (M.R.); 2 Department of Mechanical Engineering, College of Engineering, Imam Mohammad Ibn Saud Islamic University (IMSIU), Riyadh 11432, Saudi Arabia; amaasiri@imamu.edu.sa; 3 Department of Industrial Engineering, College of Engineering, Imam Mohammad Ibn Saud Islamic University (IMSIU), Riyadh 11432, Saudi Arabia; aaelfar@imamu.edu.sa; 4 Department of Mechanical Engineering, Faculty of Engineering, University of Tabuk, Tabuk 71491, Saudi Arabia

**Keywords:** hydrothermal, environmental contamination, imidacloprid, photocatalytic degradation

## Abstract

Imidacloprid (IMI), the commonly used neonicotinoid pesticide, has emerged as a persistent aquatic contaminant due to its high solubility and stability, posing risks to non-target organisms and ecosystem health. In this study, a MnZnFe_2_O_4_/SrWO_4_ ferrite–tungstate nanocomposite was synthesized via a hydrothermal process and its ability to photocatalytically degrade IMI under UV light was assessed. SEM, XRD and FT-IR were used to characterize the composite to confirm its structural and morphological features. Photocatalytic performance was systematically investigated by examining the effects of operational factors, including initial pollutant concentration, catalyst dosage, pH, and irradiation time. The MnZnFe_2_O_4_/SrWO_4_ nanocomposite exhibited significantly enhanced activity, achieving up to 87% degradation of IMI within 30 min at pH 9, outperforming individual components (SrWO_4_: 37%; MnZnFe_2_O_4_: 75%) under identical conditions. The degradation kinetics followed a pseudo-first-order model consistent with the Langmuir–Hinshelwood mechanism. Effective interfacial charge transfer between the ferrite and tungstate phases, which suppresses electron-hole recombination and increases the production of reactive species, is responsible for the enhanced performance. Furthermore, the composite demonstrated good stability and reusability across several cycles, indicating its practical applicability. Overall, the results demonstrate the potential of MnZnFe_2_O_4_/SrWO_4_ nanocomposites as efficient and sustainable photocatalysts for removing imidacloprid and similar organic contaminants from aqueous systems.

## 1. Introduction

Pesticide contamination in aquatic environments has become a major global concern due to the widespread and persistent use of neonicotinoids [1,2]. Among these, *imidacloprid* (*IMI*) is one of the most extensively applied systemic insecticides in agriculture, exhibiting high water solubility (0.61 g L^−1^ at 20 °C) and chemical stability (half-life up to 190 days in water and 997 days in soil) which allow it to persist and accumulate in ecosystems. In surface water and groundwater, IMI residues have been found in concentrations of 0.01–320 μg L^−1^ especially where there is high agricultural runoff. Even at low levels, trace concentrations have very dangerous effects on aquatic life: in the case of the organism, the median lethal concentration (LC50) was 2.1 mg L^−1^ in the case of *Daphnia magna*, and the levels of chronic toxicity were observed at concentrations of 1 μg L^−1^ [3,4]. It also has a low photodegradation rate (rate constant 0.004–0.012 h^−1^ in natural sunlight), which also adds to its persistence [5]. Although regulatory authorities in most jurisdictions, such as in the European Union, cannot renew the use of IMI and the U.S Environmental Protection Agency has recently prohibited the use of IMI, residual concentrations are still a significant issue within the environment, as they signal the need to find a lasting solution by removing it [6,7]. Among remediation methods, advanced oxidation processes (AOPs) based on heterogeneous photocatalysis have emerged as promising pathways for the mineralization of recalcitrant organic micro-pollutants, including IMI. Photocatalysis has the potential to convert the contaminant into CO_2_, H_2_O, and inorganic components, rather than relocating the pollutant to another phase, as would be the case with conventional biological or adsorption techniques [8]. The use of semiconductors on a ceramic platform is of particular interest in this regard because of its chemical stability, mechanical endurance, irradiation resistance, and the possibility of reuse in cyclic operation, an imperative characteristic of effective water-treatment systems [9]. Photocatalysts are widely used to drive redox processes for the synthesis of heterocyclic scaffolds under light irradiation, as well as to degrade heterocyclic pollutants such as IMI. Through photocatalytically generated radical intermediates, such processes enable the selective and sustained production of new complex heterocycles, including five-membered nitrogen-containing rings [10].

A group of ceramic photocatalysts, the so-called scheelite-type tungstates, with the general formula ABO_4_ (A Ca, Sr, Ba; B Mo, W) are of great interest to researchers due to their wide band gaps, great chemical stability, and strong crystalline structures, making them applicable in severe aqueous conditions [11]. According to recent research, Zn doping in BaMoO_4_ results in increased pseudocapacitive behavior and structural disorder, which enhances the photocatalytic capabilities of CaMoO_4_ and SrMoO_4_ materials while keeping the distinctive scheelite morphology [12]. A good example is strontium tungstate (SrWO_4_), which has a tetragonal scheelite structure; it has great photochemical stability and a large oxidative potential under ultraviolet light due to its wide band gap of about 4.1 eV [13]. Recent research on tungsten-based photocatalysts has shown that they may effectively clean oxidants under light irradiation, such as hydroxyl radicals and hydrogen peroxide, highlighting their promise for environmentally friendly water treatment [14]. However, the main disadvantage of SrWO_4_ and other wide-gap tungstates is the low absorption of visible light and the rapid rate of photogenerated electron-hole pair recombination, which reduces quantum efficiency and prevents effective operation in sunlight [15]. In line with this, it is urgent to develop strategies that improve charge separation, broaden the optical response, and enable catalyst recovery. At the same time, spinel ferrites (Mn, Co, Ni, etc.) with the overall formula M_x_Zn_1−x_Fe_2_O_4_, have been considered in photocatalytic reactions and Fenton-like oxidations. Specifically, manganese-zinc ferrite (Mn_x_Zn_1−x_Fe_2_O_4_) is both magnetic separable (high saturation magnetization, low coercivity) and tunable in its electronic structure through cation substitution to provide interfacial charge-transfer when coupled with other semiconductors [16]. As single components, ferrites act as photocatalysts, radical-generating oxidation catalysts; as part of heterostructures, they may offer charge-sink properties, magnetically reconfigurable structures, and high photo stability [17].

Prior studies on the photocatalytic degradation of IMI have involved both ferrite-based and non-ferrite catalysis. For instance, a mesoporous copper ferrite (CuFe_2_O_4_) catalyst used in a heterogeneous Fenton-type system showed a pseudo-first-order rate constant of approximately 1.0445 h^−1^ for IMI (~10 mg L^−1^) under optimal conditions [18]. Although this performance is high, full mineralization was not demonstrated, and catalyst recovery in repeated cycles remained limited. In another study, a Z-scheme heterojunction based on graphitic carbon nitride (g-C_3_N_4_) and slag-derived calcium ferrite improved IMI removal by about 2.5 times over 120 min compared with pure g-C_3_N_4_ [19]. Ferrite tungstate heterojunctions like ZnFe_2_O_4_@SrWO_4_ hollow microspheres [20] and CoFe_2_O_4_@ZnWO_4_ [21] have demonstrated promising activity in photo-Fenton dye degradation and photo-reduction via the S-scheme mechanism, but their application to persistent pesticides like IMI remains unexplored, often requires longer irradiation times (>120 min), and lacks magnetic recoverability. Similarly, the current IMI catalysts g-C_3_N_4_/CaFe_2_O_4_ Z-scheme (2.5-fold faster degradation over 120 min) [19] and the most recent DyMo@MnFe_2_O_4_ composite suffer from low UV efficiency and less reusability. Among non-ferrite systems, ZnO-based catalysts have been reported to achieve about 95% IMI degradation within one hour under UV irradiations [6]. However, these systems are often limited by UV activity only, poor magnetic separation, and reduced stability during reuse [22]. Similarly, an Ag_2_O@CuO (92% in 120 min) [23]. Ag-ZnO composite for IMI degradation, where performance improved over pure ZnO, yet visible-light efficiency remained low and long-term reusability was not reported [24]. Additionally, a composite of 10% Co_3_O_4_–MoO_3_ showed ~98% removal of IMI under natural sunlight in ~3–4 h but the catalyst stability and recyclability were not fully documented [25].

To address these limitations, this study presents a novel MnZnFe_2_O_4_/SrWO_4_ nanocomposite synthesized via hydrothermal and co-precipitation methods as an efficient photocatalyst for IMI degradation. The novelty of this work lies in the integration of magnetic MnZnFe_2_O_4_ with scheelite-type SrWO_4_ to synthesize a recoverable photocatalyst. MnZnFe_2_O_4_/SrWO4 nanocomposite achieved 87% IMI degradation within only 30 min under UV irradiation, with a pseudo-first-order rate constant of 0.047 min^−1^ outperforming individual components (SrWO_4_; 37% and MnZnFe_2_O_4_; 75%) because of synergistic heterojunction charge transfer, decreased recombination (PL quenching) and catalyst recovery after five cycles (>70 percent retention).

## 2. Experimental Details

### 2.1. Materials

Sigma-Aldrich, St. Louis, MO, USA, provided all of the reagents, which were used exactly as supplied: Strontium nitrate (Sr(NO_3_)_2_) (99%), sodium tungstate dihydrate (Na_2_WO_4_·2H_2_O) (99%), Potassium hydroxide (KOH) pellets (90%), manganese nitrate tetrahydrate (Mn(NO_3_)_2_·4H_2_O) (98%), Zinc nitrate hexahydrate (Zn(NO_3_)_2_·6H_2_O) (≥98%), Iron (III) Nitrate Nonahydrate (Fe(NO_3_)_3_·9H_2_O) (98%), polyvinylpyrrolidine (PVP, surfactant) (99%), ethanol C_2_H_5_OH (99.8%) and Confidor (17% imidacloprid) were bought from the agricultural crop company “Bayer”, Monheim am Rhein, Germany. Deionized water (2.0 MΩ/cm) was used throughout.

### 2.2. Synthesis of Strontium Tungstate (SrWO_4_)

**Figure 1:**
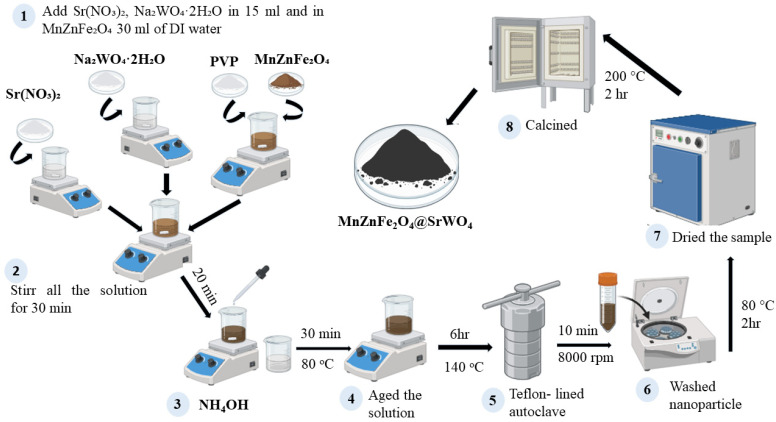
Schematic representation of the preparation of MnZnFe_2_O_4_/SrWO_4_. nanocomposite via a hydrothermal-assisted co-precipitation method, showing sequential formation and integration of MnZnFe_2_O_4_ and SrWO_4_. phase.

Strontium tungstate (SrWO_4_) nanoparticle synthesis was performed using a co-precipitation approach as shown in Figure 1 [26]. Initially, two separate 0.40 M solutions were prepared by dissolving 1.27 g of Sr(NO_3_)_2_ and 1.98 g of Na_2_WO_4_·2H_2_O in 15 ML of deionized water under moderate stirring at ambient temperature. This corresponds to a stoichiometric Sr^+2^ and WO_4_^−2^ molar ratio of 1:1, ensuring complete scheelite phase formation [27]. The strontium solution was then added dropwise into the tungstate solution under vigorous stirring. The reaction mixture was heated to 70 °C and stirred continuously for 3 h, while the pH was adjusted and maintained at 8.0 with aqueous KOH to ensure uniform nucleation. This led to the formation of fine white precipitate. After 3 h, the suspension was left to age for 12 h at ambient temperature to allow particle growth. The resulting precipitate was then centrifuged at 5000 rpm and washed repeatedly with deionized water until the supernatant was clear. The resulting solid was dried at 80 °C to remove surface moisture, then calcined at 200 °C for 2 h in a muffle furnace, yielding well-crystallized SrWO_4_ nanoparticles.

### 2.3. Synthesis of Manganese Zinc Ferrite (MnZnFe_2_O_4_)

MnZnFe_2_O_4_ nanoparticles were synthesized via a PVP-assisted co-precipitation method. Stoichiometric amounts of metal nitrates, i.e., 2.5 mmol Mn(NO_3_)_2_ (0.717 g), 2.5 mmol (ZnNO_3_)_2_·6H_2_O (0.743 g), and 10.0 mmol Fe(NO_3_)_3_·9H_2_O (4.040 g), were separately dissolved in deionized water and diluted to 25 mL each. The solutions were combined under vigorous stirring, and PVP (0.1%) was used as a stabilizer. The mixture was heated to 80 °C, and precipitation was induced by dropwise addition of concentrated KOH solution to maintain pH. The suspension was aged at 80 °C for 3 h to promote crystallite growth. The product was isolated by centrifugation, repeatedly washed with deionized water and ethanol until neutral pH was reached, and dried at 80 °C for 12 h. Finally, the powder was calcined at 200 °C for 2 h (5 °C/min ramp) to decompose organics and enhance crystallinity, yielding phase-pure MnZnFe_2_O_4_ nanoparticles.

### 2.4. Synthesis of MnZnFe_2_O_4_/SrWO_4_

The MnZnFe_2_O_4_/SrWO_4_ nanocomposite was synthesized [20] by weighing MnZnFe_2_O_4_ (0.50 g) and dispersed in deionized water (30 mL) containing PVP (1 wt%) and sonicated at (50 °C, 15 min). PVP was added as a stabilizer and surface-directing agent. Separate 0.10 M solutions of Sr(NO_3_)_2_ (15 mL) and Na_2_WO_4_·2H_2_O (15 mL) were adjusted to pH 8.5. During vigorous stirring at 80 °C (600 rpm), the Sr^+2^ and WO_4_^−2^ solutions were added dropwise to the ferrite dispersion over 20 min, with the pH maintained at 8 by adding NH_4_OH. The mixture was aged 30 min at 80 °C, transferred to a Teflon-lined autoclave, and heated at 140 °C for 6 h. It optimizes interfacial bonding while preserving ferrite crystallinity [28]. The solid was recovered by centrifugation (8000 rpm, 10 min), sequentially washed with DI water and ethanol until the supernatant was neutral, and dried at 80 °C overnight.

Upon completion, the product was collected by centrifugation at 8000 rpm for 10 min, then washed several times with deionized water and absolute ethanol until a neutral supernatant was obtained. Finally, the purified composite powder was dried at 80 °C overnight, yielding a fine, homogeneous MnZnFe_2_O_4_/SrWO_4_ powder.

### 2.5. Photocatalytic Degradation of Imidacloprid (IMI)

The photocatalytic degradation mechanism of imidacloprid using the synthesized MnZnFe_2_O_4_/SrWO_4_ nanocomposite is presented in Figure S1. Ultraviolet irradiation was supplied by a mercury lamp (emission wavelength: 365 nm; Konrad Benda Herolab, Wiesloch, Germany) with a power rating of 8 W and an irradiance intensity in the range of 10–12 mW cm^−2^ across an illuminated area of 42.75 cm^2^. The light source was positioned 10 cm above the reaction system. Photocatalytic experiments were performed under UV exposure, while control studies in the absence of illumination were conducted to assess non-photocatalytic effects. Aqueous imidacloprid solutions were prepared using commercial Confidor within a concentration range of 10–50 mg L^−1^. In a typical experiment, 100 mL of a 30 mg L^−1^ solution was used and maintained at ambient temperature (25 °C). Before irradiation, the catalyst was introduced into the solution at concentrations ranging from 0.1 to 0.6 g L^−1^ and stirred magnetically at approximately 400 rpm in the dark for 10 min to ensure adsorption–desorption equilibrium. Following this, the suspension was exposed to UV light while continuous stirring was maintained throughout the reaction. At specific time intervals, 3 mL aliquots were withdrawn and analyzed using a UV–visible spectrophotometer at 269 nm (Figure S2) to determine the residual imidacloprid concentration. The photocatalytic degradation efficiency was subsequently calculated using Equation (1).

(1) $$Degradation\;efficiency\%=1-\frac{C}{CO}\times 100$$

where C denotes concentration at a particular time interval and C_0_ represents initial concentration. This study examines the catalytic efficiency using a variety of experimental parameters, such as photodegradation time, catalytic concentration (0.1–0.6 g/L), pesticide dosage (10–50 ppm) and pH (3, 5, 7, 9 and 11). The pH of the solution can be adjusted using 0.1 M KOH and 0.1 M HCl. The following formula is employed in the kinetic model to calculate the photocatalyst’s kinetic behavior and rate constant: ln (C/C_0_) = k_app_ × t, where C_0_ is the initial concentration and C is the concentration at a given time interval. The natural logarithm of C/C_0_ is represented by ln (C/C_0_) and the apparent rate constant (*K_app_*), which includes both the adsorption of Imidacloprid on the catalyst surface and the following photoreaction upon UV light irradiation, is the total degradation rate under pseudo-first-order kinetics. It measures how quickly the substance’s concentration drops over time, illustrating the combined effects of degradation, light intensity and the catalytic efficiency. The higher the value of (*K_app_*), the faster the reaction proceeds. It also provides information about the reaction order: t denotes the reaction time. The following formula can be used to determine the reaction’s half-life and rate constant: T_1/2_ = 0.693/k_app_, where T_1/2_ or half-life denotes the amount of time needed for the concentration.

The term ln(C/C_0_) represents the natural logarithm of the ratio between the instantaneous concentration (C) and the initial concentration (C_0_). The apparent rate constant (k_app_) describes the overall degradation rate following pseudo-first-order kinetics, incorporating both the adsorption of imidacloprid onto the catalyst surface and its subsequent photochemical transformation under UV irradiation. This parameter reflects the rate at which the pollutant concentration decreases over time, accounting for the combined influence of catalytic activity, light intensity, and reaction conditions. A higher value of k_app_ indicates a more rapid degradation process. The variable *t* corresponds to the reaction time.

The half-life (T_1_/_2_) of the reaction, defined as the time required for the pollutant concentration to decrease to half of its initial value, can be determined using the relation

T_1_/_2_ = 0.693/k_app_,

where k_app_ is the apparent rate constant.

For kinetic evaluation based on the Langmuir–Hinshelwood (L-H) kinetic model, the initial linear segment of the ln(C/C_0_) versus time plot was considered. The slope of this region provides the value of k_app_, and the initial reaction rate (r_0_) was subsequently calculated using Equation (2).

(2) r_o_ = K_app_ C_o_

The L-H model shown in Section 5.6 used these r_o_ values.

### 2.6. Characterization of Structure, Morphology and Optics

The concentration of imidacloprid in solution was monitored using a UV–visible spectrophotometer (Specord 200 Plus, Jena, Germany) over the wavelength range 190–1100 nm. The maximum absorption peak (λ_max_) for the commercial Confidor formulation (containing 17% imidacloprid, a neonicotinoid pesticide) was identified at 269 nm, as illustrated in Figure S2. At predetermined time intervals, 3 mL aliquots were withdrawn from the reaction mixture to measure absorbance.

The crystalline phases of the synthesized photocatalysts were characterized by X-ray diffraction (XRD) using an X’Pert3 MRD diffractometer (Malvern Panalytical, Malvern, UK) equipped with a Cu Kα radiation source, with data collected over a 2θ range of 10–80°. Surface morphology was examined using scanning electron microscopy (SEM) and elemental composition was determined by energy-dispersive X-ray spectroscopy (EDS); the corresponding results are summarized in Table S1.

## 3. Results and Discussion

### 3.1. Optical Property

**Figure 2:**
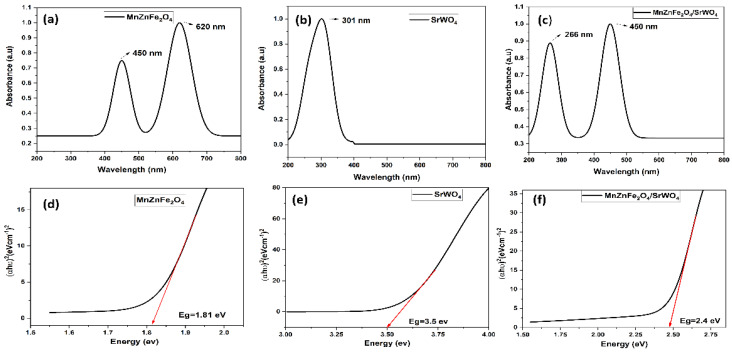
UV–Vis spectra (**a**–**c**) and their corresponding Tauc plots (**d**–**f**) of MnZnFe_2_O_4_, SrWO_4_, and MnZnFe_2_O_4_/SrWO_4_ nanocomposite, illustrating their optical absorption behavior and their energy bandgap.

The optical behavior of MnZnFe_2_O_4_, SrWO_4_, and MnZnFe_2_O_4_/SrWO_4_ nanostructures was investigated using UV–Vis absorption spectroscopy, and their corresponding band gaps were evaluated using the Tauc method. In Figure 2, the absorption spectra of all three samples are indicated in the wavelength range of 200–800 nm. A unique absorption edge is observed in each sample, indicative of its inherent electronic structure and transition properties. The MnZnFe_2_O_4_ nanoparticles exhibited two distinct absorption peaks at 450 nm and 620 nm, which are assigned to O^2−^(2p) → Fe^3+^(3d) ligand-to-metal charge-transfer (LMCT) transitions and weaker d–d crystal-field transitions of Fe^3+^ and Mn^2+^ ions occupying octahedral and tetrahedral sites, respectively. The fact that the XRD pattern does not show Fe^2+^ means that no intervalence (Fe^2+^/Fe^3+^) transition is involved in the absorption; rather, the high intensity of the visible absorption is due purely to permitted ligand-to-metal charge transfer (LMCT) transitions as well as to defect-related surface states. The following Equation (3) can be used to determine the interaction between the incident photon energy and the excess energy available to create electron-hole pairs in a semiconductor.

(3) (αhν)n = A(hν − E_g_)

where α is the absorption coefficient; hν is the photon energy (eV), which is the sum of the incident light (ν) and Planck’s constant (h); Eg is the optical band gap. A stands for constant and n represents the quantity of electron-hole pairs formed. The direct optical band gap (E_9_ = 1.8 eV) obtained from the Tauc plot, as shown in Figure 2, corresponds to electronic excitation from the O 2p valence band (HOMO) to the Fe 3d conduction band (LUMO). The fact that the band gap is relatively narrow with a high absorbance intensity is suggestive of efficient π → π* and n → π* transitions aided by centrally coordinated Fe^3+^. This extensive visible absorption corresponds to a bathochromic (red) shift compared to standard bulk ferrites, which is a consequence of strain on the lattice, redistribution of cations and increasing the orbital overlap (because of the nanoscale effect), which lowers the excitation energy and increases the absorption edge into the visible spectral range. Therefore, MnZnFe_2_O_4_ is an optically sensitive Fe^3+^ controlled semiconductor in which Fe^2+^ does not play a role. By comparison, the SrWO_4_ nanostructures exhibited a single and strong absorption edge at 301 nm, which is typical of a wide-band-gap scheelite-type oxide. The XRD findings were in line with the tetragonal phase of high crystallinity and zero impurity reflection. The UV absorption is based on the O 2p → W 5d (n → π*) charge-transfer transition, i.e., it is an excitation of electrons in the oxygen non-bonding (HOMO) orbital to tungsten antibonding (LUMO) orbital. The Tauc plot yielded a direct band gap of 3.5 eV, corresponding to intrinsic SrWO_4_, and confirmed strong UV absorption and little activity in the visible region. The big HOMO-LUMO gap restricts optical excitation to larger photon energies, and this is the reason why the material is transparent in the visible spectrum. SrWO_4_ has a distinctly hypsochromic (blue) shift at its absorption edge compared to MnZnFe_2_O_4_ (both of which have a smaller band gap and no defect-induced states), indicating a highly ordered electronic structure with minimal subband gap transitions.

The MnZnFe_2_O_4_/SrWO_4_ composite is a hybrid optical material comprising ferrite and tungstate transitions. The UV–Vis spectrum shows two large absorption bands, one of which is 266 nm, which is the O 2p → W 5d (n → π*) transition of the SrWO_4_, and the other one is 450 nm, the O 2p → Fe ^3+^ (3d) (π → π*) transition of the ferrite material. This Tauc analysis, based on the indirect band gap (E g = 2.4 eV), falls between those of the parent oxides, indicating orbital hybridization between Fe^3+^ (3d) and W^6+^ (5d) states at the interface. This hybridization produces shallow electronic states at the conduction band minimum, essentially lowering the excitation energy and increasing absorption in the visible light. The emergence of the lower-energy absorption edge relative to pure SrWO_4_ represents a bathochromic (red) shift, indicating that interfacial electronic coupling and charge delocalization reduce the overall band gap. The redshift also establishes a good transfer of charges across the Fe-O-W junction, with electrons excited in the O 2p HOMO state in the ferrite material being able to transfer to the hybrid Fe/W 3d-5d LUMO state, which facilitates prolonged light collection. This composite therefore becomes a combination of the high UV response of SrWO_4_ and visible absorption of MnZnFe_2_O_4_ to create a photoactive material of a wide spectrum.

### 3.2. Functional Group Analysis

**Figure 3:**
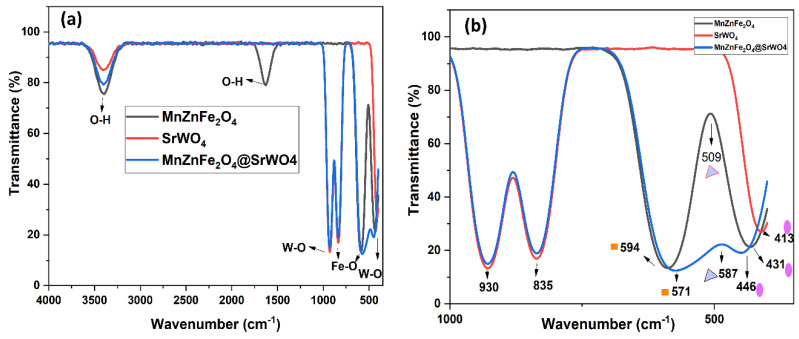
FT-IR analysis of MnZnFe_2_O_4_, SrWO_4_, and MnZnFe_2_O_4_/SrWO_4_ shown in (**a**) full range and (**b**) the 1000–400 cm^−1^ region, confirming the characteristic metal oxygen vibrational modes of both phases.

The FTIR spectra in Figure 3 clearly reflect the vibrational features of both MnZnFe_2_O_4_ and SrWO_4_ and MnZnFe_2_O_4_/SrWO_4_. Figure 3b demonstrates the FTIR spectra of nanocomposites with wave numbers between 400 and 950 cm^−1^. A significant portion of the spectra is the vibrational region between 400 and 1000 cm^−1^, which can be attributed to molecular vibrations of the metal–oxygen bond in the photocatalysts. For the ferrite sample, two characteristic absorption bands are observed in the lower frequency region. The strong band at 560 cm^−1^ corresponds to the stretching vibration of metal–oxygen bonds at the tetrahedral sites, while the band near 430 cm^−1^ arises from the octahedral site vibrations of the spinel lattice. These two features are typical of Mn–Zn spinel ferrites and are consistent with earlier reports [29]. The SrWO_4_ phase is confirmed by the presence of a pronounced absorption peak at 820 cm^−1^, which corresponds to the asymmetric stretching vibration of the W–O bonds in the WO_4_^2−^ tetrahedral units. This peak position agrees well with previously reported FTIR data for scheelite-type SrWO_4_ [15,30], indicating that the internal tetrahedral geometry of the tungstate group remains intact in both the pure SrWO_4_ and the composite material. Broad O–H stretching and bending modes observed at 3400 cm^−1^ and 1630 cm^−1^, respectively, originate from adsorbed water on the particle surfaces, a common feature in oxide nanomaterials. Importantly, no additional absorption bands associated with impurity phases such as Fe_2_O_3_, FeOOH, or WO_3_ are detected, indicating that the synthesized materials are phase-pure. The combination of ferrite (A–O/B–O) and tungstate (W–O) vibrational modes in the composite further supports the successful integration of both structures without chemical degradation.

### 3.3. XRD Analysis

**Figure 4:**
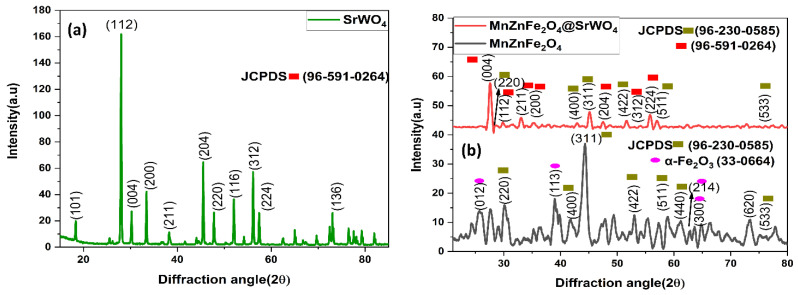
XRD diffractogram of (**a**) SrWO_4_ (green) (**b**) MnZnFe_2_O_4_ (black) and MnZnFe_2_O_4_/SrWO_4_ (red).

The crystal structure and phase purity of Mn_0.5_Zn_0.5_Fe_2_O_4_, SrWO_4_, and the SrWO_4_/MnZnFe_2_O_4_ composite were examined by powder XRD (Cu Kα, λ ≈ 1.5406 Å). The diffraction patterns are shown in Figure 4. The diffraction pattern of Figure 4a shows that SrWO_4_ powder is completely different and can be indexed to a tetragonal scheelite structure (space group I4_1_/a). Distinct peaks are observed at 2θ values typical of SrWO_4_, which we assign to the (101), (004), (200), (211), (204), (220), (116), (312), (224) and (136) planes, in agreement with the scheelite-type earlier reports on nanocrystalline SrWO_4_ with JCPDS 96-591-0264 (marked in red colored rectangle shape [15]. No additional peaks from SrO, WO_3_, or other tungstate phases are detected, confirming that the co-precipitation route followed by mild calcination at 200 °C yields phase-pure SrWO_4_ with good crystallinity. Similar diffraction behavior has been reported by Fathima et al. and by Chen et al. [31], who also observed intense (112) and (211) reflections for chemically synthesized SrWO_4_. The XRD pattern of the MnZnFe_2_O_4_ sample exhibits a series of well-defined peaks at around 2θ ≈ 30°, 35°, 43°, 53°, 57°, 62° and 74°. These reflections can be indexed to the (220), (311), (400), (422), (511), (440) and (533) planes of a cubic spinel ferrite with space group Fd3̅m. The peak positions and their relative intensities agree well with the Mn_0.5_Zn_0.5_Fe_2_O_4_ reference pattern (JCPDS 96-230-0585) reported in the literature [32,33] This confirms that the main phase formed in our synthesis is monocrystalline MnZnFe_2_O_4_. Besides the spinel peaks, a set of much weaker reflections is also visible in the MnZnFe_2_O_4_ pattern at 2θ ≈ 24°, 33°, 35.5°, 40.8°, 49.4°, 54.0°, 57.5°, 62.4°, 63.9° and 71.9°. These can be indexed to the (012), (104), (110), (113), (024), (116), (018), (214) and (300) planes of rhombohedral α-Fe_2_O_3_ (hematite) [33]. Their very low intensity relative to the spinel (311) peak indicates that hematite is present only as a minor secondary phase in the as-prepared MnZnFe_2_O_4_ powder, most likely due to partial oxidation of iron during ferrite synthesis and calcination. MnZnFe_2_O_4_ prepared via sol–gel, co-precipitation, and combustion synthesis has repeatedly exhibited similar XRD signatures in the literature [34], confirming the reliability of the structural assignment.

XRD analysis of the MnZnFe_2_O_4_/SrWO_4_ composite shows the diffractogram as superposition of the respective phases’ patterns and that the appearance of new reflections of new phases that would indicate the formation of mixed or substituted phases is absent. This finding validates that the two constituents retain their unique crystallographic arrangements. The spinel ferrite typical peaks of the (220), (311), (400), (422), (511) and (440) planes are easily recognizable to indicate that the MnZnFe_2_O_4_ phase retains a cubic crystal structure after composite synthesis. At the same time, the archetypal scheelite reflections that can be indexed as (112), (004), (200), (204), (220), (116), (312) and (224) of SrWO_4_ are also observed, which supports the fact that both materials coexist in the final specimen. The two theta values of these reflections are more or less similar to those reported in each of the individual pure phases, which indicates that there is no new bulk mixed phase present and it would be expected that the interaction between MnZnFe_2_O_4_ and SrWO_4_ is a mostly interfacial interaction. The formation of a nanocomposite is confirmed by the resulting ~16–20 reflections from both phases, which results in characteristic peak multiplicity of biphasic heterostructure, Figure 4b. The results support the development of a heterostructured composite in which the ferrite and tungstate phases retain their independent crystal structures. Similar behavior has been reported for ferrite–oxide composites, where the crystalline structures of the individual phases are retained during composite formation [20].

The minor hematite-related peaks, which could be seen in the pure MnZnFe_2_O_4_ sample, are not detectable in the composite structure. In particular, the diagnostic α-Fe_2_O_3_ reflections near 2θ ≈ 24° and 33° are absent. This disappearance can be attributed to surface encapsulation of the ferrite nanoparticles by the SrWO_4_ shell, which suppresses the diffraction contribution of any surface-bound hematite, and the reduced Fe oxidation during the composite’s hydrothermal step, where the presence of WO_4_^2−^ species offers a stabilizing chemical environment that discourages the formation of Fe–O–Fe clusters associated with hematite. Similar observations have been reported for ferrite–tungstate and ferrite–molybdate composites, where the secondary oxide layer inhibits surface oxidation [35,36].

The average crystallite sizes were estimated using the Debby Scherer Equation (4).

(4) $$D=\frac{K\lambda }{\beta \cos\theta }$$

where

K = Scherer constant (0.9);
λ = Wavelength of X-rays used (1.5406 Å for Cu Kα);
β = Full width at half maximum (FWHM) value;
θ = Angle of diffraction (2θ).

SrWO_4_ exhibits a crystalline size of 54.8 nm, MnZnFe_2_O_4_ has 34.3 nm, and MnZnFe_2_O_4_/SrWO_4_ has 16.8 nm size calculated by using Equation (2), while the percent crystallinity of MnZnFe_2_O_4_, SrWO_4_, and MnZnFe2O4/SrWO_4_ is 99.2%, 96.2% and 90%, respectively. The percent crystallinity was calculated using Equation (5). It represents how the material’s atomic arrangement is ordered with respect to the ratio of the crystalline to amorphous phases.

(5) $$Percentage\;of\;Crystalinity\;(\%)=\frac{Ac}{Ac+A\alpha }\times 100$$

where

Ac = area under crystalline peak;
Aα = area under the amorphous peak.

The XRD peaks of MnZnFe_2_O_4_ in the composite show a slight shift from 2θ = 35.5° (pure ferrite) to 35.7°, showing minimal lattice strain caused by the interfacial connection with SrWO_4_. In addition to the estimated crystallite sizes, the composite peak FWHM is wider than the individual phases. This is consistent with heterojunction formation and a smaller crystallite size (~16.8 nm). The composite crystallinity percentage drops to 90% from 92% (MZnFe_2_O_4_) and 96.2% (SrWO_4_), indicating that interface formation modifies the structure but maintains the overall crystal structures. These findings suggest that the heterojunction alters the composite’s crystallinity and lattice characteristics, thereby contributing to increased photocatalytic activity.

### 3.4. SEM and EDX Analysis

**Figure 5:**
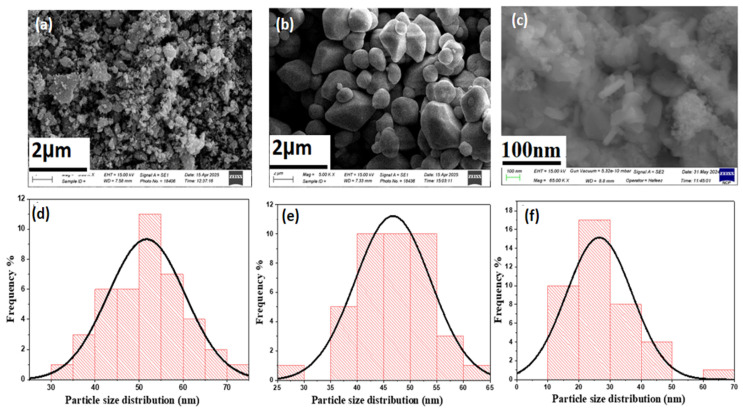
SEM images of (**a**) MnZnFe_2_O_4_, (**b**) SrWO_4_, (**c**) MnZnFe_2_O_4_/SrWO_4_ and particle size distribution of (**d**) MnZnFe_2_O_4_, (**e**) SrWO_4_, (**f**) MnZnFe_2_O_4_/SrWO_4_.

Scanning electron microscopy (SEM) was used to study the morphology and particle size distribution of the synthesized materials, revealing that each of the three samples, i.e., MnZnFe_2_O_4_, SrWO_4_, and the MnZnFe_2_O_4_/SrWO_4_ composite, has a different morphology and particle size distribution. The SEM image of MnZnFe_2_O_4_ (Figure 5a) shows that the particles are agglomerated clusters of approximately spherical nanoparticles. The size distribution of the MnZnFe_2_O_4_ material is between 25 nm and 40 nm, with most particles being between 30 nm and 35 nm, as represented in the corresponding graph (Figure 5d). Such a small distribution indicates that the synthesis procedure successfully generated comparatively homogeneous particles. Nevertheless, the existence of agglomeration as seen in the SEM images implies that the nanoparticles are prone to sticking to each other due to the existence of strong magnetic dipole–dipole forces, an observation that is in accord with other past experiments involving Mn-based ferrites, whereby a similar morphology of particles and moderate agglomeration was observed in MnZnFe_2_O_4_ prepared through the co-precipitation and solvothermal reactions. This kind of clustering has been highly reported in Mn-Zn ferrites due to their high saturation magnetization and surface energy [37]. Even though agglomerated, individual particle boundaries can be identified, indicating that primary crystal growth was not significantly hindered during synthesis [38]. Though agglomerated, the individual particle boundaries remain visible, suggesting that primary crystallite growth was not significantly hindered during synthesis. Figure 5b shows that the pure SrWO_4_ sample has a clear faceted morphology with grain sizes of between 35 nm and 60 nm, with an average size of 50 nm. Such polyhedral characteristics are typical of the tungstates of a scheelite type and crystallize with smooth edges as a result of the anisotropic rate of growth of WO_4_^2−^ tetrahedra [15]. The relatively homogeneous grain boundaries and well-defined edges indicate good crystallinity and are consistent with the strong diffraction peaks observed in XRD. The nucleation density in SrWO_4_ seems to be higher than in the ferrite and this is why the size distribution is somewhat wider when compared to MnZnFe_2_O_4_ [9]. A significant change in the morphology is observed in the composite MnZnFe_2_O_4_/SrWO_4_ (Figure 5c). The composite particle size distribution (Figure 5f) is significantly smaller, with a range of 5 to 30 nm, with the most frequent occurrence in the range of 15 to 20 nm. This smaller size and more homogeneous distribution indicate that the size of the SrWO_4_ shell is crucial for regulating the particle size of the MnZnFe_2_O_4_ heterostructure [39]. The observed reduction in the MnZnFe_2_O_4_/SrWO_4_ composite particle size ~17 nm suggests that SrWO_4_ may be involved in regulating ferrite development. This is likely due to adsorption of WO_4_^2−^ species on the MnZnFe_2_O_4_ surface during hydrothermal synthesis. This limits particle fusion and promotes uniform nucleation. Smaller grain size effects have been reported in other oxide-tungstate hybrid systems [40] in which tungstate and molybdate phases are used to alter the growth kinetics [41]. The smaller particle size in the composite means there are no large, phase-segregated particles representing ferrite or tungstate. The absence of discrete, isolated domains implies that the composite cannot be a simple physical mixture and may be aggregated into separate domains [42]. The reduction from 28 nm (MnZnFe_2_O_4_) and 49 nm (SrWO_4_) to 17 nm (MnZnFe_2_O_4_/SrWO_4_) supports this explanation. Surface area is usually increased by finer particles, which in turn increases photodegradation.

**Figure 6:**
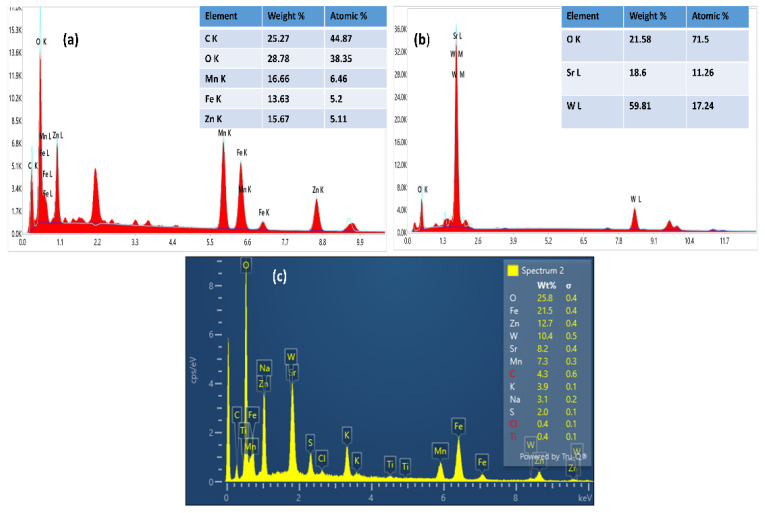
EDX spectra of (**a**) MnZnFe_2_O_4_ (**b**) SrWO_4_ and (**c**) MnZnFe_2_O_4_/SrWO_4_. Confirming the successful incorporation of the expected elemental composition.

Figure 6a–c shows the EDX spectra of the synthesized materials that prove the presence of all the expected elements. In the case of MnZnFe_2_O_4_, the formation of the spinel ferrite is confirmed by the formation of distinct peaks of Mn, Zn, Fe and O without any impurity phases. A small carbon signal is visible, attributable to residual surfactant from the low-temperature synthesis with CTAB; this is consistently observed and has no negative effect on phase purity. The SrWO_4_ sample exhibits strong Sr, W, and O peaks with an almost perfect Sr:W ratio, indicating that the scheelite phase has formed successfully. In the MnZnFe_2_O_4_/SrWO_4_ composite, the simultaneous presence of Mn, Zn, Fe, Sr, W, and O confirms the coexistence of both phases. No extra elemental peaks were observed, demonstrating that the composite is chemically pure and that the synthesis process did not introduce any unwanted species. The XRD study nearly supports the EDS analysis of the photocatalysts.

### 3.5. Photoluminescence (PL) Analysis

**Figure 7:**
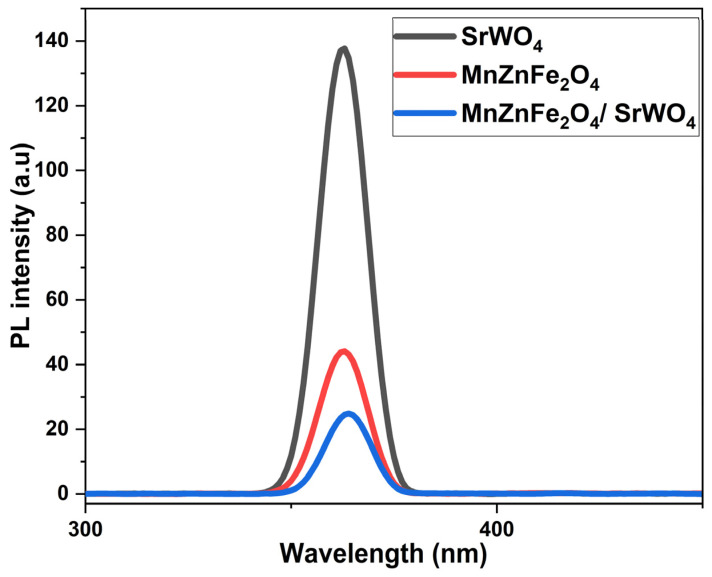
PL spectrum of the MnZnFe_2_O_4_/SrWO_4_ photocatalyst in comparison with the MnZnFe_2_O_4_ and SrWO_4_ reduced PL spectra (blue), enhancing charge separation and suppressing photogenerated electron-hole recombination.

Photoluminescence (PL) studies of synthesized nanomaterials MnZnFe_2_O_4,_ SrWO_4_, and MnZnFe_2_O_4_/SrWO_4_ are shown in Figure 7 to investigate the charge carrier recombination behavior. Data show a sharp peak for MnZnFe_2_O_4_ and SrWO_4_ at 362 nm, attributed to the intrinsic emission from the electronic transition of ionized oxygen vacancies, cationic disorder in the spinel structure of MnZnFe_2_O_4_, and the scheelite-type structure of SrWO_4_. These charge-transfer transitions of Fe^+3^/Mn^+2^ → O^−2^ and O^−2^ → W^+6^ promote radiative recombination, which leads to poor photocatalytic performance. However, the composite of MnZnFe_2_O_4_/SrWO_4_ peak slightly shifted to 364 nm with dramatically quenched PL intensity, indicating efficient interfacial charge transfer and suppressed photo carrier recombination. Furthermore, compared to individual MnZnFe_2_O_4_ and SrWO_4,_ the MnZnFe_2_O_4_/SrWO_4_ composite has a smaller semicircle in the Nyquist plot, as shown in Figure S3. According to Electrochemical Impedance Spectroscopy (EIS), lower charge transfer resistance and more effective interfacial hole separation demonstrate that the heterojunction promotes electron-hole separation. This ultimately favors photocatalytic activity.

## 4. Photocatalytic Degradation Mechanism of Imidacloprid Using MnZnFe_2_O_4_/SrWO_4_ Catalyst

**Figure 8:**
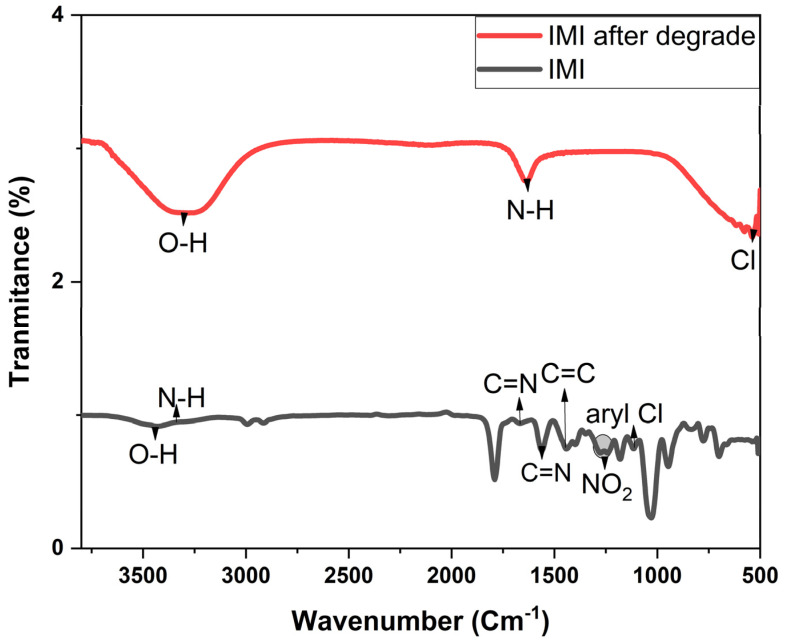
FTIR spectrum of the product produced by imidacloprid photodegradation compared to the pristine imidacloprid, demonstrating significant changes in functional group vibrational bands (red).

Light absorption, charge separation, and surface redox reactions all contribute to the synergistic heterojunction-driven photocatalytic degradation of imidacloprid over the MnZnFe_2_O_4_/SrWO_4_ composite. MnZnFe_2_O_4_ and SrWO_4_ both generate electron–hole pairs when exposed to UV light. At the MnZnFe_2_O_4_/SrWO_4_ junction, a built-in interfacial electric field is established due to the favorable band alignment between the two semiconductors. This field prevents charge carriers from recombining and promotes their directional migration [6]. Photogenerated electrons preferentially accumulate in the MnZnFe_2_O_4_-rich region’s conduction band, where they react with dissolved oxygen to produce superoxide radicals (•O_2_), which can further transform into H_2_O_2_ and ultimately produce hydroxyl radicals (•OH), which are highly reactive. Photogenerated holes move toward the SrWO_4_-rich region at the same time and directly oxidize surface-adsorbed water molecules or hydroxide ions to produce additional •OH radicals [25]. The predominant oxidative agents that are responsible for attacking the imidacloprid molecules that are adsorbed on the surface of the catalyst are these reactive oxygen species, particularly •OH. The radicals attack imidacloprid’s electron-rich sites first, such as the nitroimine group, heterocyclic rings, and the chloropyridinyl moiety. This causes stepwise bond cleavage, ring opening, and the formation of intermediate fragments that are gradually mineralized into benign end products such as CO_2_, H_2_O, and inorganic ions (e.g., NO_3_^−^/NH_4_^+^ and Cl^−^). Overall, the heterojunction architecture’s effective interfacial charge separation, extended carrier lifetime, and increased generation of reactive oxygen species all contribute to MnZnFe_2_O_4_/SrWO_4_ composite being a promising photocatalyst for environmental remediation, especially in pesticide-contaminated water. To verify the photodegradation process, FTIR spectroscopy was employed in Figure 8.

The imidazoline ring structure is evidenced by the N-H stretching vibration at 3338 cm^−1^ in the pristine IMI spectra (black), indicating that this group is intact in IMI prior to degradation. The nitrile (C≡N), NO_2_ group (NO_2_) and conjugated C=C bonds inside the pyridine ring. The presence of these bonds is responsible for the pesticide’s reactivity. C=N and C=C stretching bands seen at 1558 cm^−1^, 1236–1277 cm^−1^ and 1439 cm^−1^. Furthermore, the chlorinated aromatic ring in IMI is reflected by the aryl chlorine signal at 1108 cm^−1^. Prior to degradation, these linkages are essential parts of the imidacloprid structure. Several prominent changes are observed after photocatalytic degradation (red spectrum). The most prominent shift is the presence of the O-H stretching band at 3423 cm^−1^, which is a clear indicator of hydroxyl group formation. This is probably because the imidacloprid molecule is being attacked by reactive oxygen species (ROS) such as hydroxyl radicals (•OH). The new peak overlapping with the N-H band at 1620 cm^−1^ indicates changes in the imidazoline ring due to hydrolysis or ring cleavage during degradation. The disappearance of C=N, C=C and NO_2_ stretches indicates the breakdown of these functional groups. Following degradation of IMI, spectra observe peaks at 3423 cm^−1^ for H-O-H stretching and bending vibration due to the aqueous medium, while N-H stretching at 1620 cm^−1^ indicates detection of the byproduct [43].

**Figure 9:**
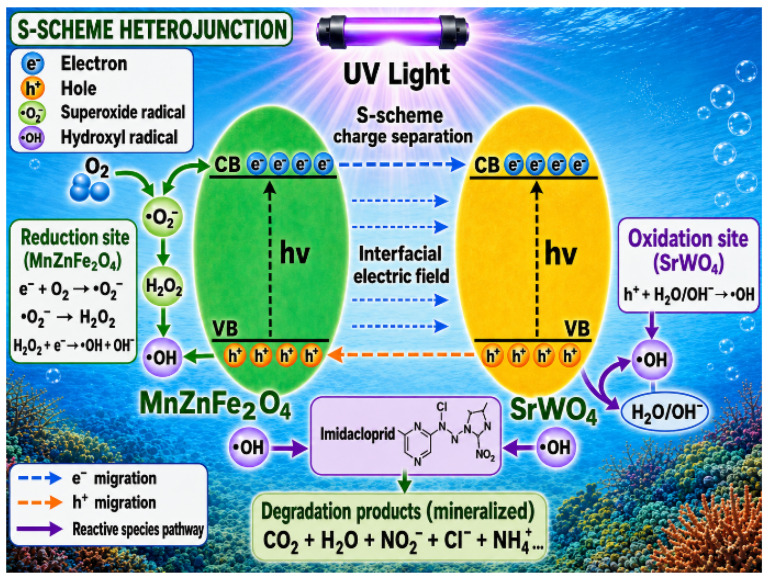
Schematic diagram illustrating the proposed photocatalytic degradation mechanism of imidacloprid mineralization via photoexcitation, charge separation, and generation of ROS using the MnZnFe_2_O_4_/SrWO_4_ photocatalyst under UV light.

The proposed heterojunction charge-transfer mechanism shown in Figure 9 is based on the observed photocatalytic enhancement and interfacial charge-transfer indicators such as reduced PL and lower EIS. Therefore, the suggested Type-II model remains tentative and further investigations of the band alignment will be carried out.

The following Equations (6)–(11) provide a complete picture of the green photocatalytic degradation of the imidacloprid pollutant using MnZnFe_2_O_4_/SrWO_4_.

1. Light sensitization:
  (6) Light + MnZnFe_2_O_4_/SrWO_4_ → e^−^ + h^+^
2. Redox Process
  (a) Reduction Site (electron warehouse: MnZnFe_2_O_4_)
    (7) e^−^_CB_ + O_2_ → •O_2_^−^
    (8) •O_2_^−^ ↔ •HO_2_ → H_2_O_2_
    (9) H_2_O_2_ + e^−^ → •OH + OH^−^
    Zn^+2^ + e^−^ → Zn^0^
    Photo-Fenton reaction
    Fe^+2^ + H_2_O_2_ → Fe^+3^ + •OH + OH^−^
    Mn^+2^ + H_2_O_2_ → Mn^+3^ + •OH + OH^−^
  (b) Oxidation site (holes factory: SrWO_4_)
    (10) h^+^_VB_ + H_2_O/OH^−^ → •OH + h^+^
3. Removal of Pollutant
  (11) C_9_H_10_ClN_5_O_2_ + •OH → CO_2_ + H_2_O + NO_2_^−^ + Cl^−^ NH_4_^+^

### Scavenger Study

Scavenger experiments were conducted to clarify the role of reactive species in the photodegradation of IMI. Ethylenediamine (EDTA) and Ascorbic Acid (AA) were used as scavengers for photogenerated holes (h^+^) and reactive species (primarily •OH), respectively. The test was conducted under the same irradiation conditions with an initial IMI concentration of 30 mg L^−1^. The degradation efficiency was 87% when scavengers were not present. The degradation efficiency dropped to 49% when EDTA was introduced as a h^+^ scavenger. When AA was added as a ROS scavenger, the degradation efficiency decreased to 62%, which is 24% less than that of control as shown in Table 1 and Figure S4. These findings show that ROS (mostly ·OH) and photogenerated holes (h^+^) both play a major role in the photocatalytic degradation of IMI especially (h^+^). The mechanistic theory drawn from the PL spectroscopy results in Figure 7 is directly supported experimentally by these scavenger data.

## 5. Imidacloprid Degradation Study

Imidacloprid photocatalytic degradation was investigated by optimizing time, catalytic concentration, pH, dosage of pollutant and comparative evaluation of catalytic efficiency parameters.

### 5.1. Photocatalytic Efficiency Comparison

**Figure 10:**
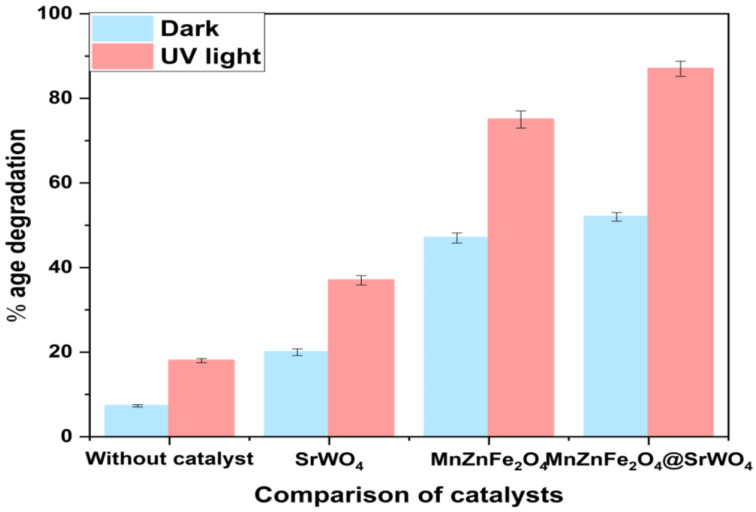
Comparative percentage degradation efficiency of MnZnFe_2_O_4_, SrWO_4_ and MnZnFe_2_O_4_/SrWO_4_ catalysts in dark and UV light experimental conditions to evaluate the effect of charge separation and synergetic interaction between two phases.

Figure 10 shows the comparative degradation efficiencies of Imidacloprid in the absence of any catalyst and in the presence of SrWO_4_, MnZnFe_2_O_4_ and MnZnFe_2_O_4_/SrWO_4_ nanocomposite under dark conditions for 24 h and under ultraviolet (UV) for 30 min. The photocatalytic efficiency of different catalysts varies with their composition and light-absorbing properties. In this catalyst comparison, a 30 ppm Imidacloprid solution was used with 20 mg of photocatalyst at pH 9. In the absence of a catalyst, the system degrades by only about 7% in darkness (increasing to 18% under UV light; however, this confirms the very low role of direct photolysis in Imidacloprid removal). Pure SrWO_4_ exhibits low activity with dark and UV removal of about 20 and 37 percent, respectively. This low performance is due to its wide band gap (approximately 3.5 eV), which limits photon absorption and promotes rapid electron-hole recombination, despite its good crystallinity.

MnZnFe_2_O_4_ is more active (approximately 47 percent in the dark and approximately 75 percent under UV light). Its narrower band gap (~1.8–1 eV) leads to greater effectiveness in light absorption and charge-carrier generation. However, partial nanoparticle agglomeration (~28 nm) is observed by SEM, which limits access to surface-active sites and hence the overall catalytic efficiency. The highest degradation rate is obtained with the MnZnFe_2_O_4_/SrWO_4_ nanocomposite, reaching values of about 52 and 87, respectively, in darkness and under UV radiation. Moderate dark remediation is explained by an increase in surface area due to a decrease in particle size (~17 nm). The radical improvement in the heterojunction structure is supported by the significant improvement observed when the structure operates under UV light. X-ray diffraction confirms the coexistence of phases, and no contaminant is formed; the optimal separation band gap (2.4 eV) and strong Fe-O-W interfacial connectivity promote effective charge separation. The suppressed recombination, as evidenced by photoluminescence quenching, increases the carrier lifetime and enhances the generation of reactive oxygen species.

The optimization of band structure, the transfer of charge caused by the heterojunction, and the refinement of nanoscale morphology are all directly correlated with the degradation trend of MnZnFe_2_O_4_/SrWO_4_ > MnZnFe_2_O_4_ > SrWO_4_ > no catalyst. Therefore, the high performance of the composite can be explained by the synergistic combination of structural integrity, optimized electronic properties, and improved surface reactivity, making the composite the best photocatalyst for degrading Imidacloprid under UV irradiation.

### 5.2. Time Exposure’s Impact on Imidacloprid’s Photodegradation Efficiency

**Figure 11:**
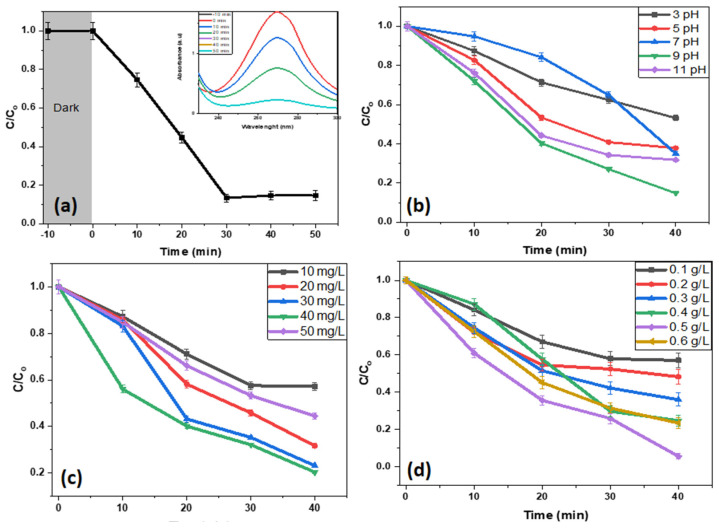
Influence of key parameters (**a**) irradiation time (0–50 min), (**b**) pH (3–11), (**c**) IMI concentration (10–50 mg/L), and (**d**) catalyst loading (0.1–0.6 g/L) on photocatalytic degradation efficiency using MnZnFe_2_O_4_/SrWO_4_.

The influence of irradiation time on the photocatalytic degradation of IMI was studied by dispersing 0.2 g L^−1^ MnZnFe_2_O_4_/SrWO_4_ of imidacloprid (30 mg L^−1^), subsequently followed by continuous stirring for 50 min at pH 9. Figure 11a shows that under UV light exposure, degradation occurred up to an extent of 87% within half an hour. It is noteworthy that the degradation rate increased significantly during the first 30 min due to the formation of reactive oxygen species and charge separation; thereafter, a slight or statistically insignificant degradation was observed, either due to the complete breakdown of IMI molecules or the restricted availability of active sites [44]. All of the data are an average of three independent replicates (n = 3) with error bars showing the standard deviation that can be attributed to normal experimental variability.

### 5.3. Impact of pH on Imidacloprid Photodegradation

The impact of pH on the photocatalytic degradation of imidacloprid (30 mg/L) using 0.2 g/L MnZnFe_2_O_4_/SrWO_4_ under UV light is depicted in Figure 11b. The pH of the solution was adjusted to 3, 5, 7, 9 and 11 using 0.1 M HCl and NaOH. The electrostatic interactions among the catalyst surface, charged radicals, and pollutant molecules can be affected by pH variations, thereby altering the effectiveness of photocatalytic degradation. Under UV light, the observed maximum degradation % occurred at pH 5, 9, and 11, which were higher than the degradation efficiencies at pH 3 and 7 during the first irradiation stage (15 min), suggesting that slightly acidic to alkaline environments are favorable for the initial stage of the reaction. With an increase in irradiation duration (30–60 min), a noticeable difference is observed. Optimal degradation activity occurs at pH 9, where about 87% removal was obtained in 30 min. This fact proves that pH 9 is the best condition for photocatalytic degradation. This increased performance at pH 9 can be attributed to increased concentration of OH radicals. OH radicals can target the electron-deficient sites of Imidacloprid. At this point, the NO_2_ electron-withdrawing group present in IMI associates with the imidazolidine ring, inducing a partial positive charge on the adjacent –C=N, rendering it more susceptible to nucleophilic attack by the OH^−^ ionic group to fasten the process of hydrolysis [45]. Consequently, at pH 11, it shows minimal degradation efficiency owing to excessive generation of OH^−^, which, in turn, repels and prevents the reactive radicals from interacting effectively with the pollutant particles [23]. Under acidic conditions (pH 3), the efficiency of degradation is relatively low, probably because of reduced production of hydroxyl radicals and possible recombination of charge carriers in the proton-rich medium.

### 5.4. Imidacloprid Dosage Effect on the Photodegradation of Imidacloprid

The impact of commercially available Confidor (Imidacloprid) concentration governed by mass transfer and concentration gradient was evaluated from 10–50 ppm treated with 0.1 g MnZnFe_2_O_4_/SrWO_4_ catalyst under pH 9 in Figure 11c. The rate of degradation shows a definite dependence on pollutant concentration and reaction time. The IMI sequestration efficiency for the MnZnFe_2_O_4_/SrWO_4_ catalyst increased with increasing initial dosage up to 40 ppm, then gradually decreased; in contrast, the data showed an optimum at 40 ppm, with a removal rate of 80%. This efficient performance is driven by accessibility of surface active sites. However, after the optimal point, at high concentration, pollutant loading increases in the solution, leading to diffusion limitation and more competition for the scarce active site, which in turn restricts the generation of hydroxyl ions and radical play a pivotal role in photodegradation [23,46]. In addition, saturation of the solution is responsible for the deactivation of sites due to slow diffusion of IMI molecules from the catalytic surface [47,48]. Based on the final degradation efficiency, the performance of different Imidacloprid dosages (10–50 ppm) are in the following order: 40 ppm (80%) > 30 ppm (76%) > 20 ppm (68%) > 10 ppm (57%) > 50 ppm (56%). On the other hand, as the concentration is increased further to 50 ppm, the degradation efficiency is low as compared to that at 40 ppm. With that great amount of pollutants loaded, the amount of Imidacloprid molecules exceeds the active sites available on the surface of the catalyst. Since the reactive oxygen species (•OH, •O_2_^−^) generation is regulated by a constant load of catalysts, light intensity, and the time taken to irradiate the sample, the generation of radicals is constant. Taking this into consideration, a concentration of pollutants that is too high leads to an inadequate availability of radicals for full degradation. Also, an increase in concentrations can facilitate the presence and concentration of intermediate species on the catalyst surface, thus inhibiting active sites and lowering photocatalytic performance [49].

### 5.5. Initial Catalytic Concentration for Imidacloprid Degradation

The degradation efficiency of IMI is shown in Figure 11d, evaluated over the range of 0.1 g/L to 0.6 g/L of the MnZnFe_2_O_4_/SrWO_4_ catalytic composite added to a 10 ppm solution of Imidacloprid under UV light. The degradation efficiency progressively increases from 0.1–0.5 g/L. This increase is explained by the increased availability of active surface sites, which leads to more efficient adsorption of imidacloprid molecules and to an increase in the formation of photogenerated electrons and holes. This means that if the active size increases, there will be greater generation of reactive oxygen species (ROS; ∙OH and ∙O_2_^−^), thereby increasing the oxidative degradation of the pollutant. However, a significant decrease is observed when the MnZnFe_2_O_4_/SrWO_4_ concentration is further increased to 0.6 g/L. The following is the order of catalysts photocatalytic efficacy at various dosages:

0.5 g/L (94%) > 0.6 g/L (76%) > 0.4 g/L (75%) > 0.3 g/L (64%) > 0.2 g/L (52%) > 0.1 g/L (43%)

Removal efficiency of pollutant primarily influenced by presence of active sites on the catalyst surface. However, beyond a certain threshold, the decrease in degradation is attributed to nanoparticle agglomeration and the impending penetration of photon flux (e^−^ and h^+^), as well as reduced light penetration due to increased turbidity [50,51]. Furthermore, active sites become saturated, reaching equilibrium, leading to the desorption of organic pollutants.

The dark degradation behavior of IMI under slightly varied circumstances over different time scales is depicted in Figure 10 and Figure 11. Since there is no irradiation, the observed decrease in IMI concentration in the dark is primarily attributed to IMI adsorption onto the photocatalyst surface rather than photocatalyst destruction. Variations in contact time, initial IMI concentration, catalyst loading and solution Ph, which affect the adsorption equilibrium. Negligible IMI loss in the dark was verified by catalyst-free control studies, suggesting that surface adsorption is the main cause of the decline. The adsorption of IMI onto the MnZnFe_2_O_4_/SrWO_4_ surface is strongly influenced by the surface charge of a photocatalyst. It varies with pH. The zeta potential distribution at pH 9 shows a narrow peak around −25 mV, indicating a uniform surface charge and excellent colloidal stability, as shown in Figure S5. This negative charge promotes stable dispersion of the composite, which enhances its interaction with neutral imidacloprid molecules through hydrogen bonding (between surface –OH groups and IMI nitrogen atom) and dipole–dipole interactions.

### 5.6. Kinetic Analysis of Reaction

**Figure 12:**
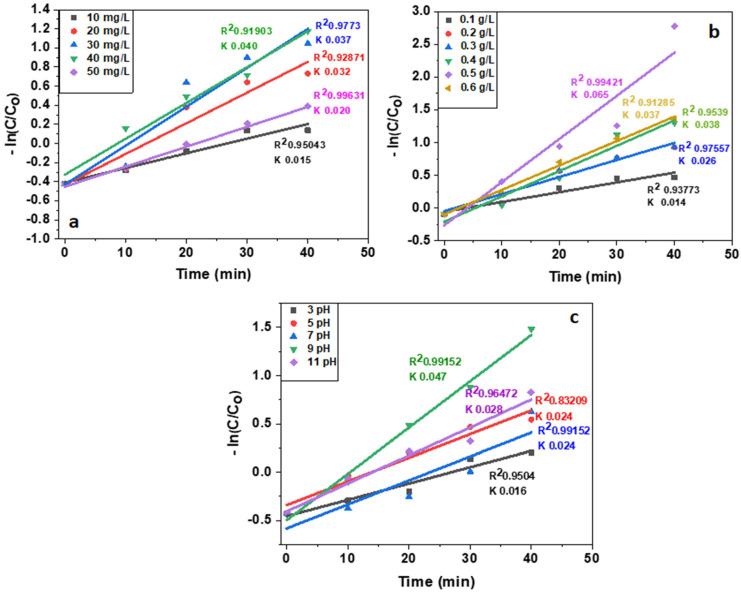
Kinetic study of photocatalytic degradation of target pollutant illustrating effect of (**a**) IMI dosage, (**b**) catalytic concentration, (**c**) pH of solution.

IMI degradation by photocatalysis is observed over an irradiation period of 30 min ^−1^ h by plotting a linear regression line ln (C/Co) vs. t; thus, the system followed first-order reaction kinetics in Figure 12a–c. The degradation rate constants of 10, 20, 30, 40, and 50 ppm were 0.015 min^−1^, 0.032 min^−1^, 0.037 min^−1^, 0.04 min^−1^ and 0.02 min^−1^, so 40 ppm showed the most efficient results with a half-life of 17.3 min. Furthermore, the removal of the pollutant in the presence of 0.1 g, 0.2 g, 0.3 g, 0.4 g, 0.5 g and 0.6 g catalysts revealed that the fitted rate constants (K) were 0.014 min^−1^, 0.026 min^−1^, 0.026 min^−1^, 0.038 min^−1^, 0.065 min^−1^ and 0.037 min^−1^. Under light exposure, the value of K increases to 0.065 min^−1^, corresponding to a half-life of 10.66 min, which decreases to 18.7 min as the reaction rate accelerates to 0.037 min^−1^. This pattern in the fitting rate constant indicates that excessive use of the MnZnFe_2_O_4_/SrWO_4_ nanocomposite induces a light-shielding effect, reducing in the removal efficiency of IMI. As the pH of the solution increases from 3 to 11 in Figure 11c, a linear relationship is observed, with fitting kinetics (K) of 0.016 min^−1^, 0.024 min^−1^, 0.024 min^−1^, 0.047 min^−1^ and 0.028 min^−1^. The study reveals the role of H^+^ and OH^−^ in the photocatalytic degradation mechanism. It is obvious to select pH 9 with the quite low half-life of 14.7 min for the follow-up experiment in basic conditions due to enhancement of OH^−^, which is involved in nucleophilic attack on the imine (C=N) or (N-NO_2_) of the imidazolidine group. Pseudo-first order was also confirmed by using a linear plot of the Langmuir–Hinshelwood (L-H) kinetic model (1/r0 vs. 1/Co) to analyze the heterogeneous surface of the MnZnFe_2_O_4_/SrWO_4_ catalyst under UV light in Equation (12). In the Langmuir–Hinshelwood model, the adsorption equilibrium constant (K) represents the affinity of the photocatalyst surface for IMI molecules. A higher adsorption at the catalyst surface is indicated by a higher K value. This raises the local substrate concentration near reactive sites, thereby accelerating degradation. A higher K value indicates stronger adsorption at the catalyst surface.

This kinetic model reveals that the entire mechanistic description comprises pollutant adsorption at active sites obtained by stirring the reactant solution for 10 min in the dark, followed by interaction of IMI with the catalyst’s photogenerated charge carriers, and finally desorption of the degraded pesticide.

(12) $$\frac{1}{r_{0}}=\frac{1}{ҡ}+(\frac{1}{ҡK})\frac{1}{C_{0}}$$

**Figure 13:**
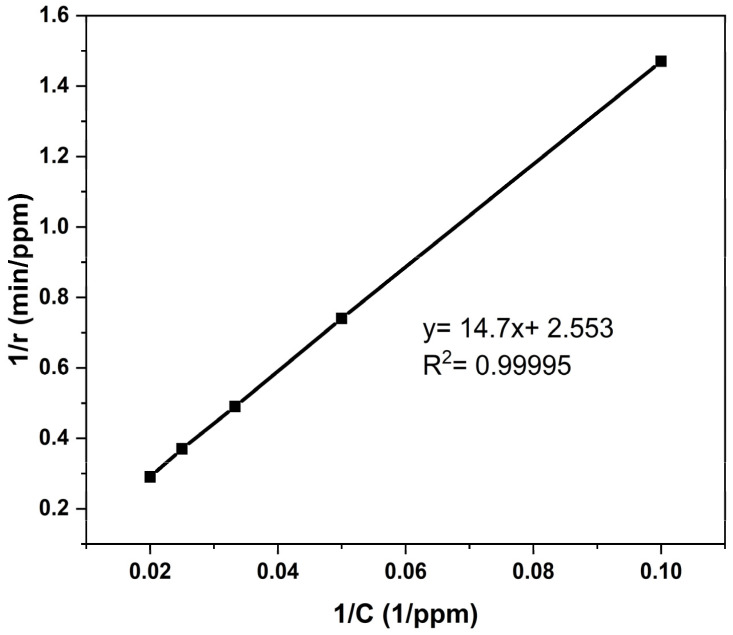
Imidacloprid photocatalyst degradation using MnZnFe_2_O_4_/SrWO_4_ photocatalyst via Langmuir Hinshelwood Kinetic fit under UV light at pH 9.

The linear plot yields an apparent rate constant (Kapp) of 14.7 min, with an excellent correlation coefficient (R2 = 0.99), as shown in Figure 13. However, the intrinsic surface reaction constant ҡ (0.06 mg/L min^−1^) and moderate adsorption affinity value of K (5.8 L^−1^ mg). This relatively high Langmuir adsorption and favorable surface reaction demonstrate efficient catalytic adsorption and surface reactivity, confirming its stability for degradation.

A concentration of 30 mg L^−1^ IMI was chosen for this work to simulate concentrated agricultural runoff and enable kinetic analysis within the optimum irradiation time. It has been reported that IMI concentrations in contaminated water sources reach up to 320 µg L^−1^ [3]. To ensure quantifiable degradation rates and enable performance comparisons among catalysts, elevated concentrations are frequently employed in photocatalysis investigations as proof of concept. With a rate constant K = 0.047 min^−1^ and good photocatalytic efficiency (87% degradation in 30 min), the MnZnFe_2_O_4_/SrWO_4_ composite demonstrated its promise as an effective catalyst for IMI degradation. However, considering that the usual range of environmental IMI values is 0.01–320 µg L^−1^ [3,52]. In order to ensure MnZnFe_2_O_4_/SrWO_4_ composites practicality, future work will focus on assessing its effectiveness at trace levels of IMI i.e., 0.01–320 µg L^−1^.

### 5.7. Reusability of the Photocatalyst for Imidacloprid Degradation

**Figure 14:**
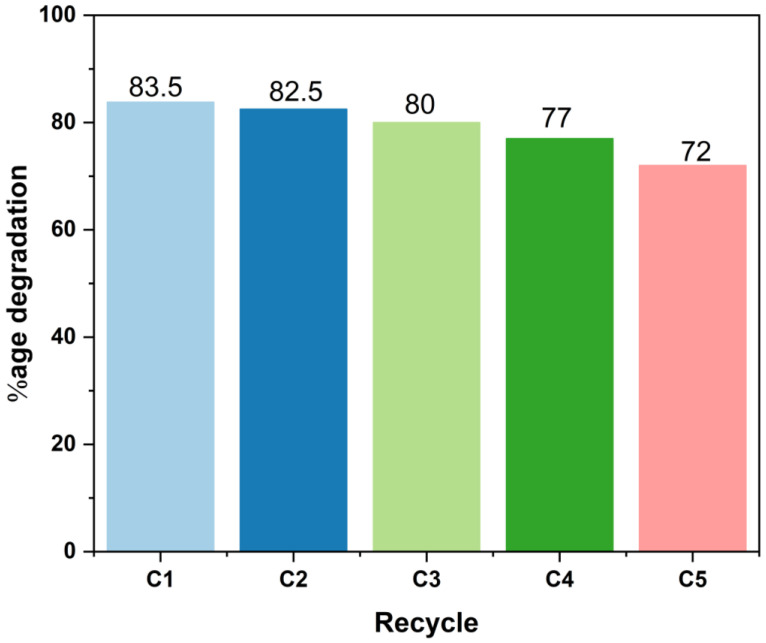
Reusability of catalyst after five consecutive cycles demonstrates retention of photocatalyst.

For the economical removal of pesticides from aqueous solutions, it is necessary to assess the catalyst’s effectiveness and chemical stability under optimal conditions. The reusability of the catalyst was evaluated over five consecutive cycles, as shown in Figure 14, yielding 72% removal at an IMI dosage of 30 ppm. The FTIR spectra of MnZnFe_2_O_4_/SrWO_4_ in the figure confirm its stability after five cycles, making it attractive and cost-effective for commercial use. At the completion of each cycle, the catalyst was recovered by centrifugation, thoroughly washed with distilled water, and dried in an oven for 1 h at 60 °C. The observed reduction in photocatalytic activity is preliminarily concerned with the loss during recovery of the catalyst, aggregation of nanoparticles, which in turn restricts sequestration and progressive fouling by intermediates [53]. A simple bar magnet can quickly separate the MnZnFe_2_O_4_/SrWO_4_ from aqueous solution as shown in Figure S6. This demonstrates the catalyst’s magnetic response; even without a quantitative assessment of saturation magnetization, the ferrite phase exhibits sufficient magnetic properties to enable effective catalyst recovery and reusability. Minor activity loss over five cycles is due to partial aggregation and surface contamination.

The MnZnFe_2_O_4_/SrWO_4_ photocatalyst FTIR spectra are shown in Figure S7 before and after five cycles. The distinctive metal–oxygen vibrations of Mn-Zn ferrite (430 and 560 cm^−1^) and the W-O peak at 820 cm^−1^, and the interfacial Fe-O-W bond remain unchanged. There is little adsorption of water or hydroxyl-containing intermediates on the surface, as indicated by slight variations in the O-H stretching area (3420 cm^−1^). The C=O, C=C and C-N stretching vibrations of adsorbed intermediate products from IMI degradation are represented by new peaks that emerge at approximately 1620 cm^−1^, 1430 cm^−1^ and 1250 cm^−1^ after five cycles. It appears that the catalyst remains chemically stable because no further notable new peaks are seen.

### 5.8. Mineralization Study of Imidacloprid

The degree of mineralization of imidacloprid after the photocatalytic degradation of Imidacloprid using MnZnFe_2_O_4_/SrWO_4_ nanocomposite was analyzed using total organic carbon (TOC) (30 0.2 g L^−1^ catalyst, pH 9). The percentage removal of the TOC was determined using the following Equation (13):

(13) $$\%\;TOC\;removal=(\frac{TOCo-TOCt}{TOCo})\times 100$$

**Figure 15:**
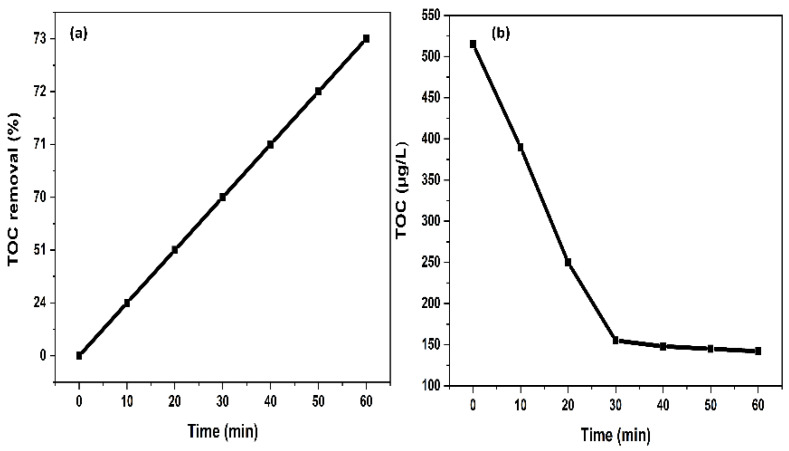
TOC measurement of a sample containing 30 mg/L of imidacloprid at pH 9 and 0.2 g/L of MnZnFe_2_O_4_/SrWO_4_ photocatalyst under 30 min of UV light. (**a**) Percentage of TOC elimination in relation to time. (**b**) TOC in μg/L in relation to time.

TOC_0_ is the TOC prior to UV irradiation, whereas TOC_t_ represents the TOC at a certain irradiation time. Figure 15 shows the removal of Imidacloprid (30 ppm/L) at pH 9 with 0.2 g/L of photocatalyst after 30 min in the UV light using a Total Organic Carbon Analyzer (TOC) (Bk-TOC (1500), Jinan, China). The initial batch of imidacloprid solution, with 100 mL at time zero, had 515 µg/L of total carbon. After 10 min of UV exposure, the TOC was reduced to 390 µg/L, equivalent to a mineralization level of 24%. Further irradiation reduced the TOC to 250 µg/L (51% removal) in 20 min.

There was a strong decrease in the first 30 min during which the TOC was 155 µg/L and this was almost the same as 70%. After 30 min, the decrease in TOC was slight, indicating that most of the mineralization had already occurred. The TOC values at 40, 50, and 60 min were 148 µg/L (71 percent), 145 µg/L (72 percent) and 142 µg/L (73 percent), respectively. The TOC results indicate that MnZnFe_2_O_4_/SrWO_4_ not only demineralizes the parent Imidacloprid molecules but also oxidizes organic carbon to final inorganic products. UV–Vis analysis showed 87% degradation in 30 min, with TOC removal of approximately 72%, indicating good but incomplete removal. This is because Imidacloprid is effectively mineralized into a comparatively less hazardous chemical, with little organic residue or intermediate material that may not be fully oxidized.

### 5.9. Comparison of Photocatalyst with Previously Reported Photocatalyst

Table 2 compares the photocatalytic degradation efficiency of the MnZnFe_2_O_4_/SrWO_4_ photocatalyst to other previously reported photocatalysts when exposed to UV light. After 30 min, the MnZnFe_2_O_4_/SrWO_4_ material (0.2 g/L) photodegrades Imidacloprid (30 ppm) with 87% efficiency at pH 9. It has a rate constant of 0.047 min^−1^. It has been reported by Tariq et al. [23] that Ag_2_O/CuO nanocomposites may break down imidacloprid (20 mg/L) by 92.3% after 180 min with a 0.3 g/L catalyst and a rate constant of 0.0031 min^−1^. The removal efficiency is also very high, but the degradation rate constant is much lower, so prolonged irradiation is required. Recently, Alqarni [54] achieved 98.1% photodegradation efficiency of Imidacloprid (10 mg/L) at 90 min using 0.5 g/L of TiO_2_/PHEMA (poly(2-hydroxyethyl methacrylate) catalyst with a rate constant of 0.0377 min^−1^, whereas the ZnO [6] photocatalyst has reached an efficiency of 95% at the same pollutant concentration after 60 min with a rate constant of 0.038 min^−1^. It has also been demonstrated that carbon-based systems, such as CDs-QDs/CQDs, can degrade imidacloprid (200 mg L^−1^) at a rate of 0.021 min^−1^ with an 85% degradation efficiency using a 0.4 g/L catalyst. With a catalyst concentration of 1 g L^−1^, CQDsSH/CdS QDs were reported to be 92% efficient with imidacloprid (10 mg L^−1^) in 90 min; however, the rate constant of degradation was not specified. Recently, DyMo@MnZnFe_2_O_4_ showed 87% degradation in 60 min; in contrast, the 0.2 g MnZnFe_2_O_4_/SrWO_4_ nanocomposite loading in the current research work demonstrates 87% degradation of IMI under significant UV exposure for 30 min. DyMo@MnFe_2_O_4_ has a 66 nm core-shell structure, while the MnZnFe_2_O_4_/SrWO_4_ composite is smaller (~17 nm) with uniform SrWO_4_, exposing more active sites. The Fe-O –W heterojunction improves band alignment and accelerates charge separation, while PL measurements show faster electron transfer and reduced recombination. These improvements enable faster photocatalytic degradation of IMI (87% in 30 min vs. 87% in 60 min for DyMo@MnFe_2_O_4_) with comparable reusability, highlighting the novelty and efficiency of our system. Collectively, these findings highlight the advantages of the proposed catalyst in UV light response, sustainability, and practicality over previously reported catalysts. For our MnZnFe_2_O_4_/SrWO_4_ heterojunction photocatalyst, the recently reported material offers useful context. By activating peroxymonosulfate under visible light, S-scheme BiOBr/CeMo_8_O_14_/r-GO heterojunction effectively degraded levofloxacin (k = 0.035 min^−1^) [55]. Similarly, under visible light, the Z-scheme ZnFe_2_O_4_/tubular g-C_3_N_4_ system exhibited improved levofloxacin mineralization (k = 0.041 min^−1^) via PMS-assisted charge separation [56]. With a competitive IMI degradation rate constant of k = 0.047 min^−1^ under UV light without PMS activation. Our S-scheme MnZnFe_2_O_4_/SrWO_4_ composite shows effective photocatalytic activity under UV light.

## 6. Conclusions and Future Perspectives

The current investigation shows that manganese-doped ferrite–tungstate nanocomposites exhibit excellent photocatalytic performance in degrading imidacloprid under UV irradiation. The optimized catalyst dosage (0.2 g L^−1^) achieved up to 87% degradation of 30 mg L^−1^ imidacloprid within 60 min at pH 9, following pseudo-first-order kinetics with a rate constant of 0.047 min^−1^. The enhanced photocatalytic efficiency is attributed to the narrow band gap (~2.5 eV), increased surface area, and improved charge separation resulting from heterojunction formation and synergistic manganese doping, which collectively suppress electron–hole recombination.

Photoluminescence analysis further confirms reduced recombination rates and efficient generation of reactive oxygen species, contributing to improved photocatalytic activity. The nanocomposite also exhibited good stability and reusability, retaining approximately 72% of its activity after five successive cycles, along with facile magnetic recovery.

Overall, these findings highlight the potential of the developed nanocomposite as a promising and sustainable photocatalyst for wastewater treatment. Future research should focus on scalable, cost-effective synthesis; evaluation under natural sunlight; long-term stability in complex wastewater matrices; and extension to a broader range of organic pollutants to validate its practical environmental applicability.

## Figures and Tables

**Table 1:** Effect of scavenger on IMI degradation over MnZnFe_2_O_4_/SrWO_4_.

Scavengers	Quenching Radicals	Degradation %
Without scavengers	**^_^**	87%
EDTA	h^+^	49%
Ascorbic acid	O_2_**^−^**	62%

**Table 2:** MnZnFe_2_O_4_/SrWO_4_ photocatalyst’s percentage of photocatalytic degradation efficiency when compared to previously published photocatalysts for imidacloprid photodegradation.

Photocatalyst	Photocatalyst Dosage (g/L)	Light Source	Imidacloprid Concentration (mg/L)	Reaction Time (min)	Imidacloprid Removal Efficiency (%)	Degradation Rate Constant, K (min^−1^)	Refs.
Ag_2_O/CuO	0.3	UV light	20	180	92.3	0.0031	[23]
CdS QDS	0.4	LED lamp	20	120	85	0.021	[51]
TiO_2_/PHEMA	0.5	Solar irridation	10	90	98.1	0.0377	[54]
CQDs-SH/CdS QDs	1	LED lamp	10	90	92	NA	[51]
ZnO	0.2	Xenon lamp	5	240	92	0.038	[6]
Zn dopped CuSe	0.2	UV light	15	60	92	NA	[57]
DyMo@MnZnFe_2_O_4_	0.2	UV light	30	60	87	0.031	[58]
MnZnFe_2_O_4_/SrWO_4_	0.2	UV Light	30	30	87	0.047	This work

## Data Availability

The original contributions presented in this study are included in the article/Supplementary Materials. Further inquiries can be directed to the corresponding authors.

## Supplementary Materials

The following supporting information can be downloaded at: https://www.mdpi.com/article/10.3390/nano16120721/s1, Figure S1: Photocatalytic degradation of imidacloprid using SrWO_4_, MnZnFe_2_O_4_, MnZnFe_2_O_4_/SrWO_4_; Figure S2: UV absorbance spectrum of imidacloprid; Figure S3: EIS spectra of MnZnFe_2_O_4_/SrWO_4_, SrWO_4_ and MnZnFe_2_O_4_; Figure S4: Radical scavenging capacity of EDTA, Ascorbic acid and without scavenger; Figure S5: Zeta Potential of MnZnFe_2_O_4_/SrWO_4_; Figure S6: Magnetic separation of MnZnFe_2_O_4_/SrWO_4_ photocatalyst using bar magnet; Figure S7: FTIR spectra of MnZnFe_2_O_4_/SrWO_4_ before and after degradation; Table S1: Instrumental specifications used for physiochemical investigation of the photocatalysts.
