# Supplementary material for: Synthesis and Photocatalytic Performance of a Ferrite-Based Tungstate Nanocomposite for Imidacloprid Removal
Source: Nanomaterials (Basel). 2026 Jun 11;16(12):721. doi: 10.3390/nano16120721 (PMC13304741; doi:10.3390/nano16120721)
Supplement: Supplementary file 1 [file nanomaterials-16-00721-s001.zip › nanomaterials-4326948-supplementary.pdf]

Synthesis and Photocatalytic Performance of a Ferrite-Based Tungstate nanocomposite for Imidacloprid Removal

Irum Jamil [1], Abdulaziz Alasiri [2], Faisal Nawaz [1]*, Muqdssa Rashid [1], Abdullah A. Elfar [3] and Md Enamul Hoque [4]*

[1] Department of Chemistry, University of Wah, Quaid Avenue, Rawalpindi, Punjab 47040, Pakistan;

[2] Department of Mechanical Engineering, College of Engineering, Imam Mohammad Ibn Saud Islamic University (IMSIU), Riyadh 11432, Saudi Arabia;

[3] Department of Industrial Engineering, College of Engineering, Imam Mohammad Ibn Saud Islamic University (IMSIU), Riyadh 11432, Saudi Arabia;

[4] Department of Mechanical Engineering, Faculty of Engineering, University of Tabuk, Tabuk 71491, Saudi Arabia

* Correspondence: faisal.nawaz@uow.edu.pk (F.N.); mhoque@ut.edu.sa (M.E.H.)

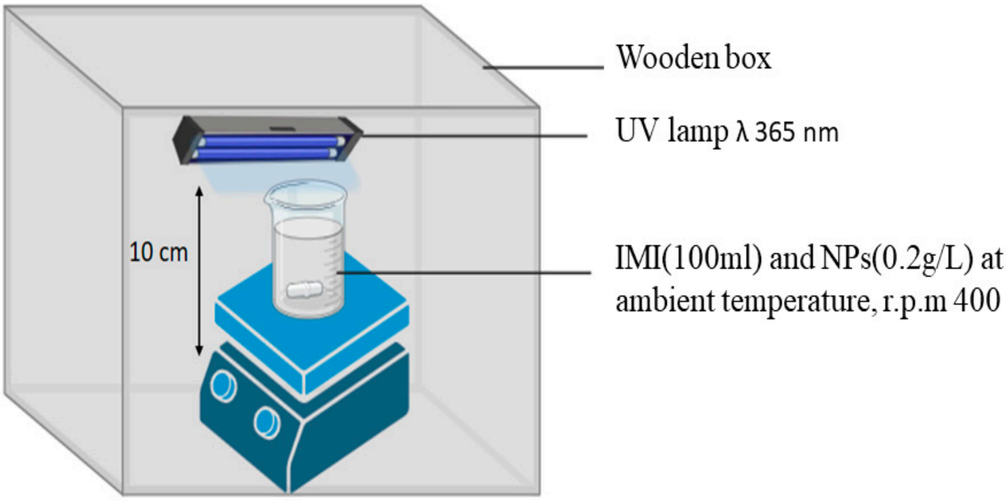

Figure S1. Photocatalytic degradation of imidacloprid using $SrWO_4$, $MnZnFe_2O_4$, $MnZnFe_2O_4/SrWO_4$

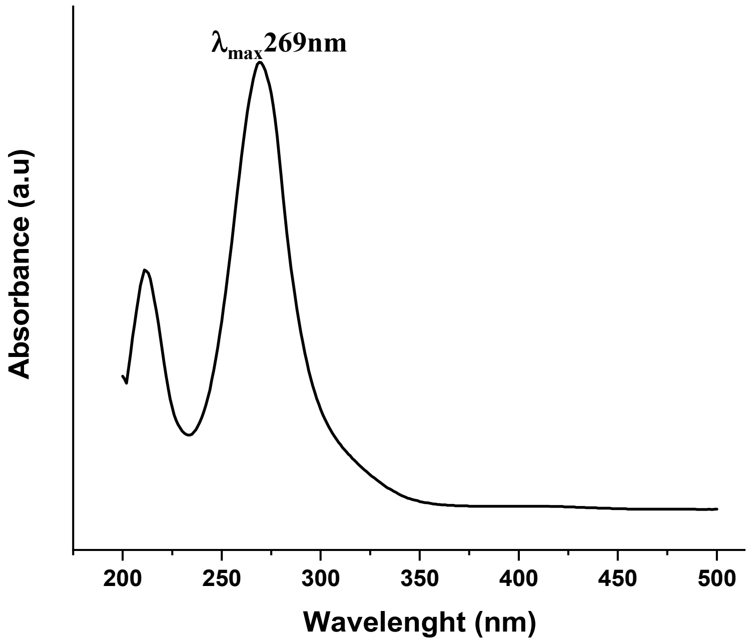

Figure S2. UV absorbance spectrum of imidacloprid.

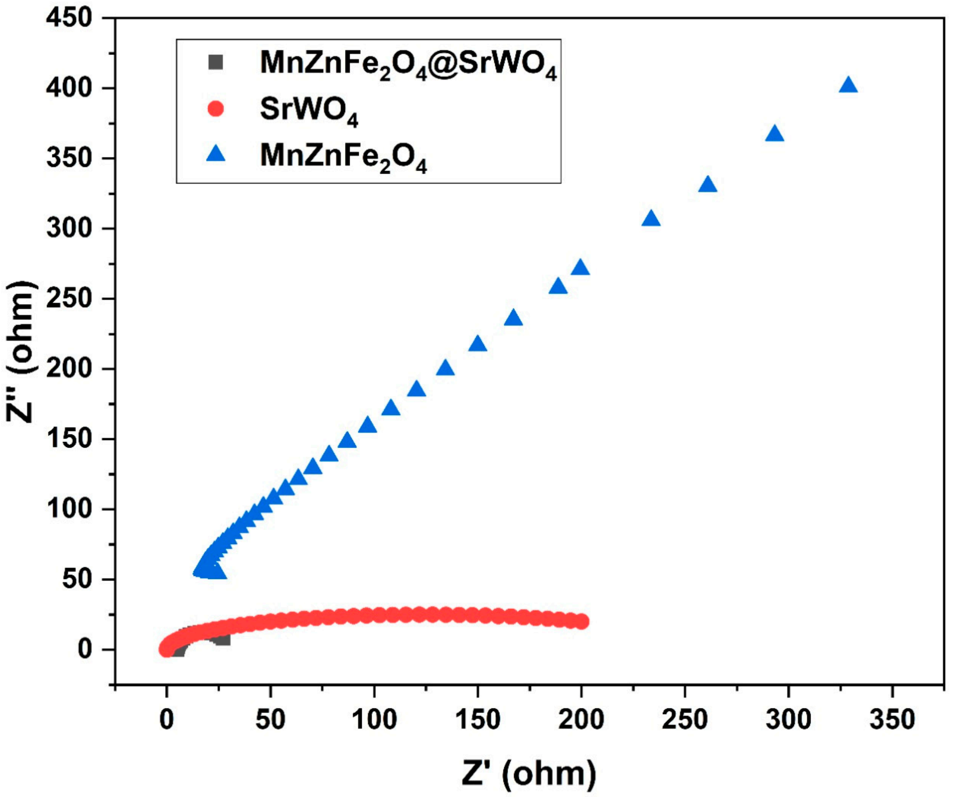

Figure S3. EIS spectra of $MnZnFe_2O_4/SrWO_4$, $SrWO_4$ and $MnZnFe_2O_4$

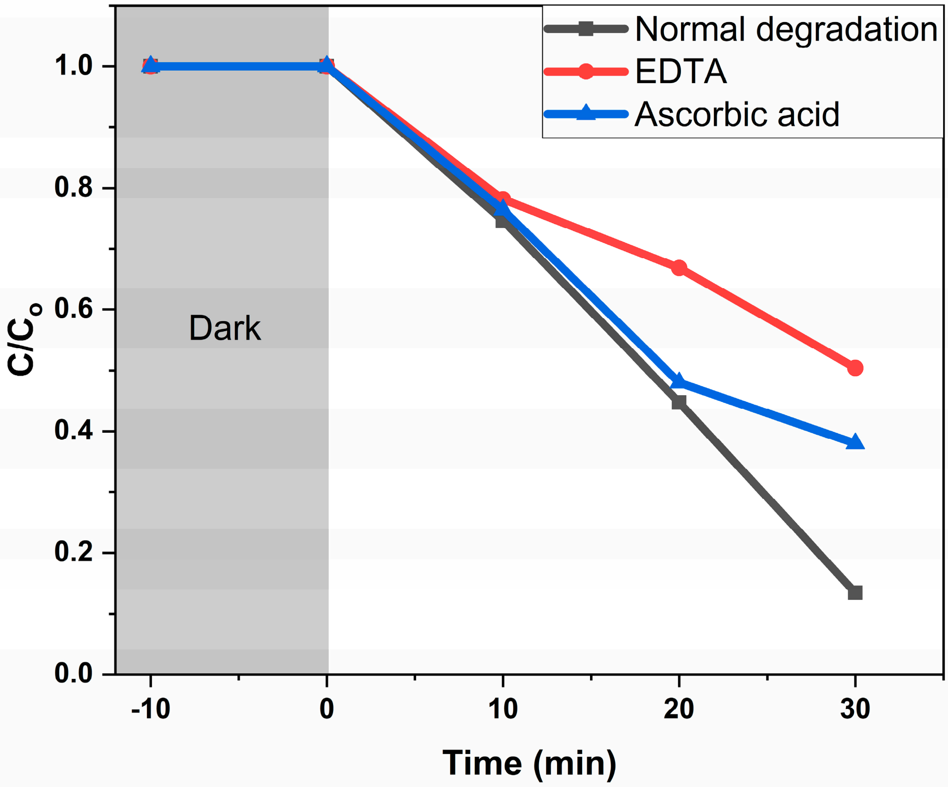

Figure S4. Radical scavenging capacity of EDTA, Ascorbic acid and without scavenger.

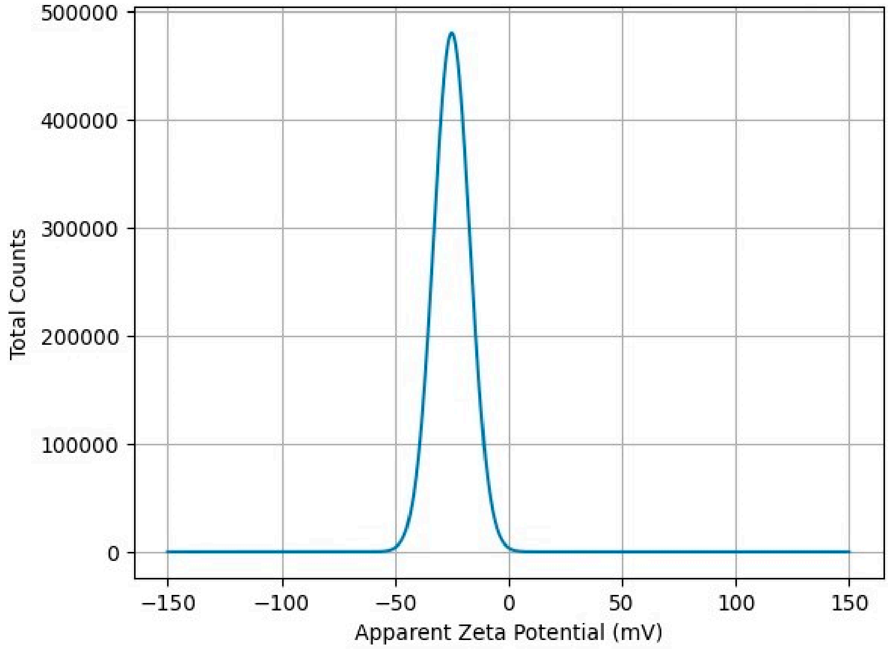

Figure S5. Zeta Potential of $MnZnFe_2O_4/SrWO_4$

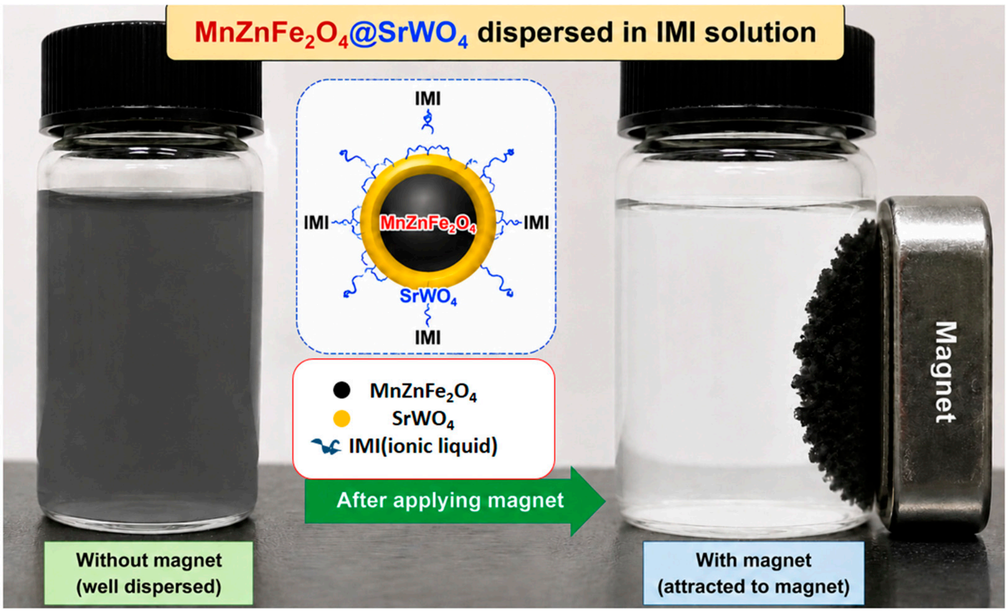

Figure S6. Magnetic separation of $MnZnFe_2O_4/SrWO_4$ photocatalyst using bar magnet

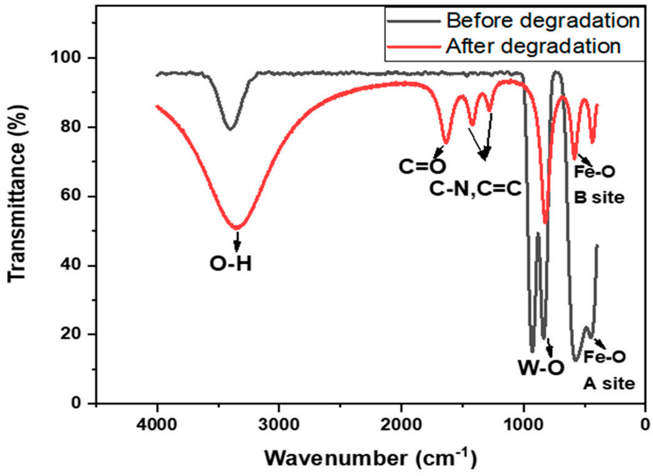

Figure S7. FTIR spectra of $MnZnFe_2O_4/SrWO_4$ before and after degradation

Table S1. Instrumental specifications used for physiochemical investigation of the photocatalysts.

**Technique**	**Instrument/ Model**	**Manufacture**	**Specification/ Operating Condition**
**UV spectrophotometer**	AI Spercord 200 Plus	Analytik Jena Germany	Double beam optical system with covering spectral range of 190 to 1100 nm, slit width: 1 nm and scan rate: 200 nm/min equipped with light source of D_2 and W- halogen lamp.
**X-Ray Diffraction (XRD)**	X'pert^3 MRD (Material Research Diffractometer)	Malvern Panalytical Netherland	Cu kα radiation source (λ_1=1.5406 A°, λ_2=1.5444 A°), Generator setting: goniometer (Pw 3050/60), scan range: 2θ (2 theta) with the scan rate of 1° min^{-1}, minimum step size omega: 0.001°, Sample Stage: reflection transmission spinner (PW 3064/60), minimum step size: phi: 0.1° with the temperature at 25 °C. slit system: divergence slit :0.5°/ receiving slit :0.1 mm.
**Photoluminescence (PL) spectroscopy**	BK-F96PRO	BIOBASE China	Having emission range 200-900 nm with the wavelength accuracy of ± 1 nm using 150 W xenon lamp.
**Fourier Transformation Infrared (FTIR) Spectroscopy**	Alpha -ATR	Bruker, Germany	Alpha –ATR spectroscopy has scan rate of 4000-400 cm^{-1}, resolution: 4 cm^{-1}, and the scan number: 32 per sample.
**Energy Dispersive X-Ray (EDX) spectroscopy**	Smart EDX	ZEISS, Germany	Fully integrated with ZEISS EVO/Sigma 300 SEM with the energy resolution of ~

	129 eV (at 1-5 nA) using Si_3N_4 window for light element x – ray with the magnification power of 5,000 x – 50,000 x.
